# Deciphering mutational effects on inducible NO synthase conformational dynamics *via* quantitative cross-linking mass spectrometry and AlphaFold2 subsampling

**DOI:** 10.1016/j.jbc.2025.110673

**Published:** 2025-10-27

**Authors:** Ting Jiang, Haikun Zhang, Gabriel Monteiro Da Silva, Yadav Prasad Gyawali, Changjian Feng

**Affiliations:** 1 Department of Pharmaceutical Sciences, College of Pharmacy, University of New Mexico, Albuquerque, New Mexico, USA; 2 Department of Molecular and Cellular Biology and Biochemistry, Brown University, Providence, Rhode Island, USA; 3 Department of Chemistry and Chemical Biology, University of New Mexico, Albuquerque, New Mexico, USA

**Keywords:** nitric oxide synthase, calmodulin, functional protein dynamics, multidomain protein, electron transfer, flavoprotein, heme, crosslinking mass spectrometry, quantitative crosslinking mass spectrometry, AlphaFold2, subsampling, conformational ensemble, conformations, timsTOF, parallel reaction monitoring

## Abstract

Mammalian nitric oxide synthases (NOSs) are flavo-hemoproteins that rely on dynamic interdomain interactions for activity. Calmodulin (CaM) facilitates specific, interdomain FMN–heme interactions that enable inter-subunit FMN–heme electron transfer essential for nitric oxide biosynthesis. Our quantitative cross-linking mass spectrometry (qXL MS) results demonstrate that the abundance of intersubunit cross-links correlates with CaM-induced formation of the docked FMN/heme complex in rat neuronal NOS [Jiang *et al.*, *Biochemistry*, 2024, 63, 1395–1411]. Here, we extend this methodology to the human inducible NOS (iNOS) isoform, comparing wild-type (wt) and E546N mutant oxygenase/FMN (oxyFMN) constructs under near-native conditions. Using parallel reaction monitoring−based qXL MS, we assessed mutation-induced changes in interdomain dynamics. The E546N mutation substantially reduced abundance of specific intersubunit cross-links between the FMN and heme domains. Cross-links between CaM and iNOS domains were also altered by the mutation, indicating that the changes at the FMN–heme docking interface propagate allosterically throughout the iNOS−CaM complex. Although standard AlphaFold2 structural modeling yielded similar docked architectures for wt and mutant, cross-link-guided AlphaLink2 modeling revealed distinct structural differences. AlphaFold2 subsampling further predicted alternative conformations; consistent with qXL MS data, E546N mutant exhibited a broader distribution of predicted conformations, with an apparent shift toward higher population of undocked states, compared to wt. Importantly, 90% of cross-links were consistent with an ensemble of representative conformations derived from AlphaFold2 subsampling and AlphaLink2 modeling, capturing both docked and undocked states alongside multiple orientations. This integrative qXL MS and AlphaFold2 subsampling strategy provides a quantitative framework for mapping mutation-induced alterations in functional dynamics of NOSs and multidomain proteins in general.

Multidomain proteins make up approximately two-thirds of eukaryotic proteomes (1, 2). These proteins are composed of modular, relatively rigid domains connected by flexible linkers/tethers, allowing for dynamic conformational motions. Such structural flexibility is essential for a wide range of biological functions, including electron transfer (ET). Efficient ET, coupled with effective enzymatic turnover, often depends on coordinated domain movements. For example, mammalian nitric oxide synthases (NOSs)—flavo-hemoproteins that produce nitric oxide—rely on both large-scale, tethered domain motions and local conformational dynamics in the docked states to facilitate the interdomain electron transfer (IET) processes (3, 4). Despite their functional importance, studying large multidomain proteins experimentally remains a daunting challenge.

Mammalian NOS is a homodimeric protein, with each subunit comprising an N-terminal heme-containing oxygenase domain and a C-terminal reductase domain (Fig. 1*A*) (5). The reductase domain consists of NADPH/FAD- and FMN-binding subdomains. The heme and FMN domains are connected by a linker containing a calmodulin (CaM)-binding sequence that forms an α-helix. In the absence of CaM, the FMN domain largely remains docked to the FAD domain, favoring IET between the FAD and FMN cofactors. NOS enzymatic activity requires CaM binding, which releases the FMN domain from its FAD–FMN docked state, enabling it to shuttle between the FAD–FMN and FMN–heme docking conformations (corresponding to the input and output states, respectively; Fig. 1*B*). These tethered FMN domain motions are facilitated by specific charged residues at the docking interfaces (4). For example, the E546 residue on the FMN domain surface of human inducible NOS (iNOS) plays an important role in forming the FMN–heme docked complex (6); pulsed electron paramagnetic resonance (EPR) studies have shown that the E546N mutation significantly decreases the population of the docked state (7). There is growing interest in developing and expanding in-solution, molecular biophysics techniques, such as hydrogen–deuterium exchange mass spectrometry (HDX-MS) (8) and pulsed EPR (9), to probe the dynamic conformational states of NOS proteins and their complexes with CaM. Despite extensive biochemical and biophysical investigations of the NOS proteins, molecular understanding of their functional protein dynamics remains incomplete, largely due to limited knowledge of the conformational landscape and how it is modulated by external cues such as CaM binding, as well as intrinsic factors like domain–domain docking interactions.

**Figure 1:**
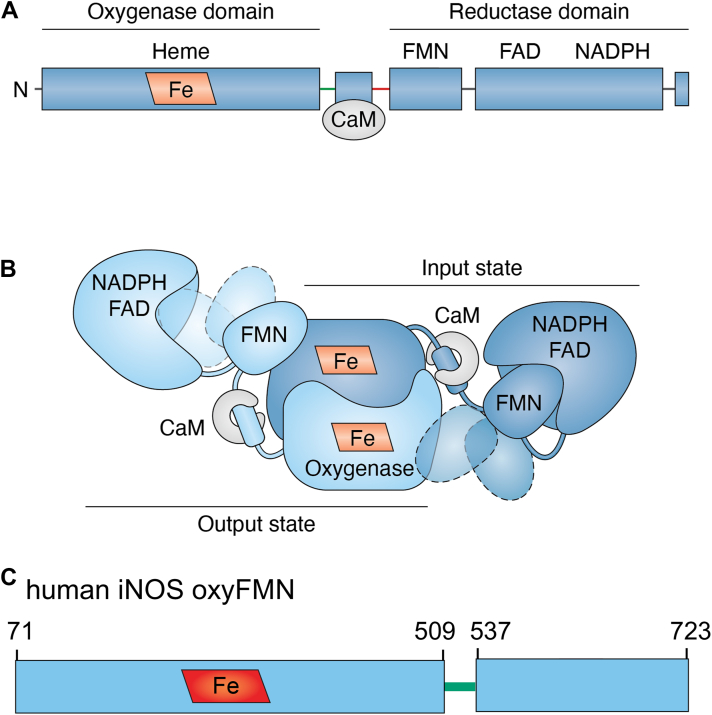
**Domain organization and conformational dynamics of mammalianNOS.***A*, the mammalian NOS isoforms are composed of an N-terminal heme-containing oxygenase domain and a C-terminal reductase domain, connected by a linker that contains the CaM-binding helix. *B*, the function of NOS relies on conformational changes triggered by CaM binding to the linker. This releases the FMN domain from the electron-accepting “input state”, enabling large-scale movements of the FMN domain that bring it into close proximity with the heme domain to form the electron-donating “output state”. The tethered FMN domain transitions between these two docking positions *via* free, undocked states (indicated by *dashed* structures). The intersubunit FMN−heme IET in the output state is essential to provide electrons to the catalytical heme iron center for NO production. *C*, schematic representation of the domains and regions in one subunit of the homodimeric human iNOS oxygenase/FMN (oxyFMN) construct. The oxyFMN protein includes a heme domain (residues 71–509), a CaM-binding linker (residues 510–536), and an FMN domain (residues 537–723). Adapted from reference (3). CaM, calmodulin; IET, interdomain electron transfer; iNOS, inducible NOS; NOS, nitric oxide synthase.

Among structural mass spectrometry techniques, cross-linking MS (XL MS) provides medium-resolution distance restraints between specific residues (10). These cross-linking data are often incorporated into integrative structural modeling approaches, such as HADDOCK (11), to simulate domain–domain docking and orientations, generating structural models of multidomain proteins. To adapt and extend these approaches, we initially employed quantitative XL MS (qXL MS) to investigate CaM binding-induced structural changes in the monomeric neuronal NOS (nNOS) reductase construct (12). Published in 2023, our original study represented the first demonstration in the NOS field of (a) using XL MS to map protein dynamics and (b) leveraging AlphaFold2 to interpret the cross-linking data (12), establishing a new framework for in-solution, comparative structural dynamics studies of these multidomain proteins and their protein-protein complexes—work that has since inspired ongoing research within the field.

We subsequently refined the strategy and developed an original workflow to examine the more intricate, homodimeric nNOS oxygenase/FMN (oxyFMN) construct (13). The bidomain oxyFMN construct (illustrated with human iNOS oxyFMN in Fig. 1*C*) provides a minimal ET model system that facilitates the study of the FMN–heme docking interactions (14, 15, 16), as it lacks the FAD domain that otherwise competes for FMN domain docking in the full-length NOS protein. The XL MS data were interpreted in the context of top-ranked AlphaFold2 models to rationalize the observed cross-links and their changes in relative abundance under varying conditions, such as CaM-binding and ionic strength (13). Our findings demonstrate that NADP^+^ ligand or CaM induces localized conformational changes near their binding sites (12). Furthermore, the abundance of specific intersubunit cross-links between the FMN and heme domains can be used as a proxy for formation of the CaM/FMN/heme docked complex (13). Other studies have also shown that XL MS can provide detailed information on interdomain interactions within the CaM–nNOS complex (17).

In this work, we investigated iNOS, another NOS isoform that produces high-output NO in response to inflammatory stimuli and plays a key role in pathogen clearance and immune regulation (18). We have extended our integrated approach of XL MS experiments and AlphaFold2 structural prediction to decipher the mutational effects on the interdomain conformational dynamics. We chose to study the E546N human iNOS oxyFMN mutant because this substitution disrupts FMN–heme interdomain docking and shifts the equilibrium between docked and undocked conformations, resulting in decreased IET rate (7). The underlying conformational dynamics are largely unclear, though. The E546N mutation was selected primarily as a mechanistic probe rather than for its association with any known disease state. Given its notably altered IET activity and docked state population, E546N serves as a valuable model to establish and validate a combined experimental and computational workflow for investigating mutational effects on docking interfaces and conformational dynamics in complex multidomain proteins.

Building on these rationales, we have conducted a detailed comparison of the relative abundance of cross-links at specific interdomain interaction sites in the wt and E546N mutant human iNOS oxyFMN proteins, with these sites initially mapped onto the top AlphaFold2 model. Recognizing that AlphaFold2 has limitations in predicting mutant structures, we also generated cross-link-guided AlphaLink2 models. Moreover, we employed AlphaFold2 subsampling approaches to predict alternative conformations. Lastly, we utilized a conformational ensemble of iNOS oxyFMN encompassing representative AlphaFold2 and AlphaLink2 models to reduce overall cross-link distance violation down to 10%. Collectively, our integrative approach enables new insights into the structural dynamics of iNOS, underscoring how the E546N mutation affects domain interactions and conformational landscapes.

## Results and discussion

**Figure 2:**
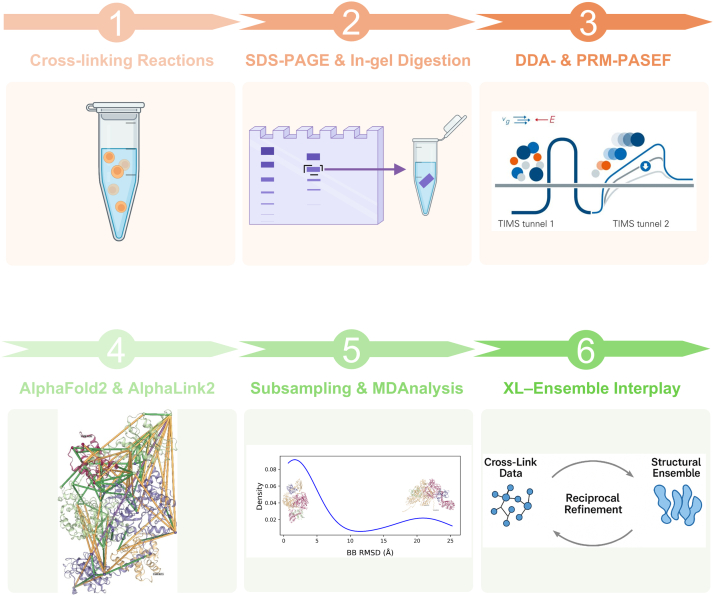
**Overall workflow for comparative protein dynamics analysis.** The DSBU cross-linking reaction conditions are first optimized to minimize overcross-linking and aggregates (step 1). The resulting cross-linked dimeric protein bands are excised, subjected to in-gel digestion (step 2), and analyzed by LC-MS/MS using DDA-PASEF mode (step 3), with replicate analyses performed under varying collision energy ramps. All unique cross-links identified are consolidated into a PRM inclusion list for targeted LC-MS/MS runs, and peptide intensities are normalized within each run (step 3). Cross-links are mapped onto *top* AlphaFold2 structural models to assign intersubunit *versus* intrasubunit pairs (step 4). Particular emphasis is on cross-links showing statistically significant differences in relative abundance between wt and mutant proteins. Experimentally observed cross-links are also incorporated as restraints in AlphaLink2 modeling to guide generation of structural models that capture domain-domain interactions and the overall architecture of the protein (step 4). In addition, AlphaFold2 subsampling is used to generate diverse conformations, followed by MDAnalysis and clustering (step 5) to identify representative structural models (*e.g.*, docked and open states, if present; representative models are illustrated in *panel 5*). Of note is that the apparent AlphaFold2 model populations do not represent thermodynamic distributions and should be viewed qualitatively. An ensemble of conformational states is then employed to reduce overall cross-link distance violations, providing a more accurate representation than a single structural model (step 6). Cross-linking captures multiple coexisting protein conformations, and evaluating cross-link satisfaction across an ensemble of structural models better reflects this conformational heterogeneity. Such reciprocal approach leverages cross-link data to guide ensemble modeling, while the ensemble reduces apparent cross-link violations by incorporating dynamic flexibility, revealing alternative structural states often missed by single models (step 6). Collectively, this integrative workflow, combining experimental, in-solution, targeted qXL MS (steps 1-3, *top panels*) and computational structural prediction (steps 4-6, *bottom panels*), is designed to provide detailed insights into the functional protein dynamics of multidomain systems, focusing on domain-domain docking interfaces. DDA, data-dependent acquisition; DSBU, disuccinimidyl dibutyric urea; LC-MS/MS, liquid chromatography-tandem mass spectrometry; PASEF, parallel accumulation-serial fragmentation; PRM, parallel reaction monitoring; qXL MS , quantitative cross-linking mass spectrometry.

Our overall workflow is illustrated in Figure 2. The targeted qXL MS experimental data are combined with AlphaFold2 predications of both top structures and conformations to map the changes in cross-links of interest to putative, regulatory domain-domain interactions that underpin efficient intersubunit FMN–heme IET in modular NOS protein.

### Intersubunit NOS-NOS cross-links in homodimeric iNOS oxyFMN protein

**Figure 3:**
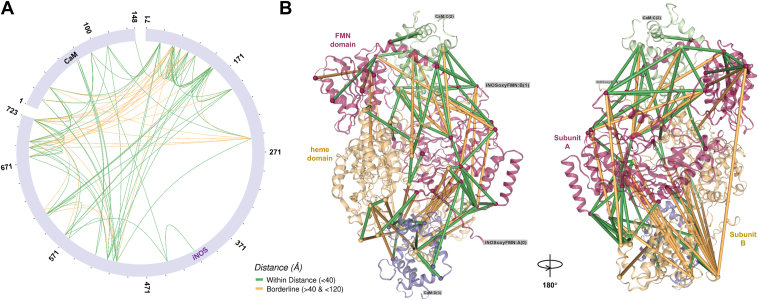
**Cross-linking and structural mapping of the iNOS oxyFMN–CaM complex.***A*, circular view of identified cross-links mapped to the top-ranked AlphaFold2 model of the wt human iNOS oxyFMN–CaM complex. Cross-links are colored based on Cα–Cα distance relative to the DSBU cross-linker threshold of 40 Å (12, 50): *green* indicates cross-links within the threshold, and *orange* indicates those exceeding 40 Å. *B*, structural mapping of the cross-links onto the AlphaFold2 model. The two iNOS subunits are shown in *red* and *light orange*, with their bound CaM modules in *green* and *purple*, respectively. The FMN domain of one subunit docks onto the heme domain of the other, supporting intersubunit FMN–heme electron transfer (57). CaM, calmodulin; DSBU, disuccinimidyl dibutyric urea; iNOS, inducible nitric oxide synthase; oxyFMN, oxygenase/FMN.

The parallel reaction monitoring (PRM) experiments detected 96 cross-links in the wt human iNOS oxyFMN−CaM complex (Fig. 3*A*); the data search was restricted to lysine residues.

We utilized top AlphaFold2 structural models to map spatial distances between cross-linked residue pairs, an emerging approach for assigning intersubunit cross-links in homodimeric proteins (13). Importantly, our top-ranked AlphaFold2 models of the wt iNOS oxyFMN–CaM complex (Fig. S1 in the Supporting Information) produce structurally plausible configurations: the predicted heme active site corresponds closely with the iNOS heme domain crystal structure (Fig. S2), even though cofactor cannot be included in the AlphaFold2 modeling. The FMN domain and CaM lobes are also arranged in a manner consistent with previous crystal structural findings (19) (Fig. S2). Leveraging these validated, high-confidence AlphaFold2 models, we then applied the "shortest-possible cross-links" criterion, along with xiVIEW visualization (13), to identify intersubunit cross-links in the dimeric CaM-bound iNOS oxyFMN protein.

Several NOS and CaM residues appear to be hyperconnected, *i.e.*, forming multiple cross-links from a single residue (Fig. 3). These hyperconnected sites are primarily located at the FMN–heme and CaM–heme(NOS) interfaces (Fig. 3*B*). The extensive yet specific nature of the docking interfaces facilitates the formation of multiple cross-links at these hotspots that are critical for forming the docked CaM/FMN/heme conformation.

We identified 26 intersubunit NOS-NOS cross-links and their sites of domain-domain interactions (Table 1). The intersubunit cross-links fall into three groups: heme–heme, heme–CaM-binding helix, and heme–FMN. When mapped onto the top AlphaFold2 model of the iNOS oxyFMN–CaM complex, all heme–heme and heme–CaM-binding helix cross-links lie within the 40 Å distance threshold. In contrast, cross-links exceeding this threshold exclusively involve the more dynamic FMN–heme interactions (see next paragraph). This pattern aligns with the established architecture of human iNOS, where dimerization is stabilized by the heme domains, resulting in a well-defined and stable inter-subunit heme-heme interface. Similarly, the CaM-binding linker remains relatively ordered in the CaM-bound state and shows no distance violations, suggesting a fairly stable orientation with respect to the homodimeric heme domains. These observations support the relative structural rigidity of the heme–heme and heme–CaM-binding helix interfaces within the dimeric iNOS protein, as captured by mapping experimental cross-linking data onto the AlphaFold2 model.

**Table 1:** Intersubunit NOS-NOS cross-links of wt human iNOS and their shortest C_α_ − C_α_ Euclidean distance mapped to the top AlphaFold2 model of human iNOS oxyFMN–CaM complex

Cross-link type	iNOS residue	iNOS site	iNOS residue	iNOS site	Shortest distance (Å)
heme − heme	88	heme	123	heme	14.36
	103	heme	113	heme	8.45
	105	heme	113	heme	5.72
	113	heme	123	heme	14.38
	113	heme	155	heme	29.08
	411	heme	445	heme	20.36
heme − CaM-binding helix	88	heme	509	CaM-binding helix	26.08
	88	heme	516	CaM-binding helix	26.11
	155	heme	516	CaM-binding helix	36.45
heme − FMN	88	heme	696	FMN	28.36
	88	heme	722	FMN	47.08
	103	heme	678	FMN	52.23
	103	heme	722	FMN	62.02
	105	heme	678	FMN	56.52
	105	heme	689	FMN	59.96
	155	heme	574	FMN	13.26
	155	heme	607	FMN	20.78
	155	heme	689	FMN	44.02
	158	heme	574	FMN	21.26
	158	heme	606	FMN	27.22
	158	heme	607	FMN	25.67
	191	heme	574	FMN	22.81
	191	heme	607	FMN	24.87
	191	heme	722	FMN	54.97
	445	heme	574	FMN	19.84
	505	heme	607	FMN	60.49

**Figure 4:**
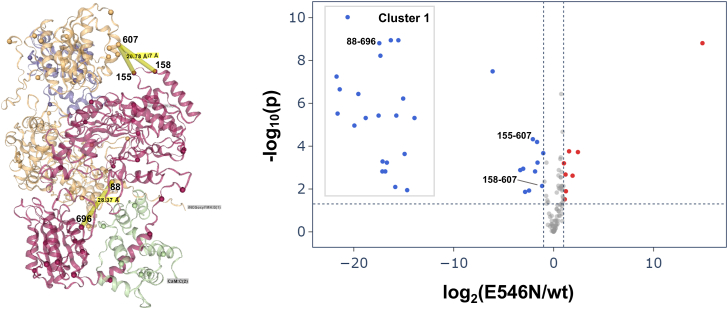
**Effect of the E546N mutation on iNOS oxyFMN–CaM cross-linking profiles.** Volcano plot illustrates how E546N mutation influences cross-link abundance profiles in human iNOS oxyFMN–CaM complex. A two-sample unpaired two-tailed *t* test was performed with the Benjamini–Hochberg correction applied. Cross-links are considered significant with an adjusted *p*-value < 0.05 and a fold-change ≥ 2. Cross-links shown in *gray* do not meet these criteria, either lacking statistical significance (*p* > 0.05) or falling below the fold-change threshold of 2. The most extreme decrease categorized as Cluster 1—correspond to cross-links detected exclusively in wt protein. These were retained for plotting by substituting zero abundance values with 1. Quantitative data represent the average of eight replicate PRM measurements. The three labeled intersubunit FMN−heme cross-links are mapped onto the top AlphaFold2 structural model of wt human iNOS oxyFMN, shown adjacent to the volcano plot. The two NOS subunits are colored *red* and *orange*, respectively. Note the intersubunit FMN–heme docking geometry. CaM, calmodulin; iNOS, inducible NOS; NOS, nitric oxide synthase; oxyFMN, oxygenase/FMN; PRM, parallel reaction monitoring.

We next focused on the intersubunit cross-links between the FMN and heme domains because the intersubunit FMN–heme domain-domain docking is crucial for the obligatory FMN−heme IET. In addition, the formation and relative abundance of the intersubunit cross-links are sensitive to the conformational states such as docked output state and undocked, free open states. We identified 17 intersubunit FMN–heme cross-links, nine of which fall within the 40 Å distance threshold (Table 1). Many of these cross-links involve lysine residues—K155 in the heme domain and K574 and K607 in the FMN domain—located at or near conserved FMN–heme docking interfaces comprising K155, K445, and R452 in the heme domain, and E546 and E603 in the FMN domain (20). Prior mutational, spectroscopic, and HDX-MS studies of iNOS have revealed charged surface residues critical for FMN–heme docking, including K445 and R452 on the heme domain and E546 and E603 on the FMN domain (6, 7, 8), where electrostatic interactions are believed to guide the docking and stabilize intersubunit contacts (8). In support of this, molecular dynamics simulations have further predicted K155 as an additional key contributor to the intersubunit docking interactions (21, 22). Here, our XL MS data provide direct experimental evidence for these predictions: K155 of the heme domain, and K574 and K607 of the FMN domain are engaged in multiple cross-links, suggesting that these regions come into close spatial proximity in the docked states. This cross-linking pattern reflects a dynamic but specific FMN–heme docking interaction, which is compatible with intersubunit FMN–heme IET. In this context, our detailed identification of intersubunit FMN–heme cross-links provides robust experimental evidence that reinforces and extends the structural features predicted by previous computational and biochemical analyses. Our XL MS results support the intersubunit FMN–heme docking topology of the iNOS oxyFMN–CaM complex (Fig. 4*B*), highlighting the role of interdomain interface in mediating the IET.

All intersubunit NOS–NOS cross-links that exceed the 40 Å distance threshold involve FMN–heme interactions (Table 1), underscoring the inherent flexibility of the FMN–heme docking interface. Most of these violations center on residues K88 and K103, with two additional outliers involving residue pairs 191 to 722 and 505 to 607. The cross-link at residue 722 exceeds the distance thresholds predicted by the top AlphaFold model, which can be attributed to flexibility at the C terminus—consistent with the low predicted local distance difference test (pLDDT) scores in this region (Fig. S1*A*).

### CaM-NOS cross-links in homodimeric iNOS oxyFMN–CaM complex

CaM is composed of two lobes (N-terminal: residues 1–75; C-terminal: residues 82–148), connected by a short flexible linker (residues 76–81). Cross-links between CaM and iNOS, along with their corresponding shortest distances in the top AlphaFold model, are listed in Table 2. CaM binds the helix (residues 510–536 in human iNOS) and also establishes extensive contacts across both the FMN and heme domains, with the majority of cross-links clustering on the heme domain (Table 2). Such interactions likely help restrict the relative motions of the FMN and heme domains, promoting ET-competent conformations. Several CaM residues, including K13, K30, K75, K77, K94, K115, and K148, form cross-linking to the NOS domains, suggesting multiple contact sites that support a broad interaction interface. These patterns are in agreement with functional regions identified through previous mutagenesis studies (8) that identified a surface of CaM encompassing R106, K115, and D118 as a key determinant of CaM–NOS docking.

**Table 2:** CaM-human iNOS cross-links and shortest C_α_ − C_α_ Euclidean distance in the top AlphaFold2 model of the homodimeric iNOS oxyFMN in complexation with CaM

CaM residue	CaM site	iNOS residue	iNOS site	Shortest distance (Å)	Subunit−Subunit^a^
13	N-lobe	88	heme	36.41	Inter
13	N-lobe	123	heme	41.36	Intra
13	N-lobe	505	heme	25.75	Intra
13	N-lobe	509	heme	20.78	Intra
30	N-lobe	88	heme	38.48	Inter
30	N-lobe	105	heme	59.22	Inter
30	N-lobe	123	heme	48.01	Intra
30	N-lobe	254	heme	50.85	Intra
30	N-lobe	505	heme	34.59	Intra
30	N-lobe	689	FMN	18.01	Intra
30	N-lobe	696	FMN	19.71	Intra
30	N-lobe	722	FMN	26.21	Intra
75	N-lobe	516	CaM-binding helix	16.31	Intra
75	N-lobe	531	CaM-binding helix	12.99	Intra
77	linker	516	CaM-binding helix	17.25	Intra
77	linker	531	CaM-binding helix	17.75	Intra
94	C-lobe	88	heme	18.56	Inter
94	C-lobe	445	heme	15.84	Inter
94	C-lobe	696	FMN	17.35	Intra
94	C-lobe	722	FMN	33.67	Intra
115	C-lobe	88	heme	20.34	Inter
115	C-lobe	254	heme	29.84	Intra
115	C-lobe	505	heme	18.95	Intra
115	C-lobe	696	FMN	21.53	Intra
148	C-lobe	103	heme	38.47	Inter

a The type of cross-link is defined by the NOS subunit that CaM is bound to. An intra-subunit cross-link occurs between CaM and its associated NOS subunit, whereas an inter-subunit cross-link occurs between CaM and the opposite NOS subunit.

Several CaM–iNOS cross-links, especially those involving CaM residue K30, exceed the 40 Å distance limit when mapped onto the AlphaFold2 model. Rather than reflecting violation, these discrepancies highlight the conformational flexibility of the docking interface that is essential for IET. Although AlphaFold2 provides a useful structural reference, it represents only a single, low-energy conformation and does not fully capture the dynamic range of motions that occur in solution. In contrast, in-solution experimental techniques like XL MS and HDX-MS directly probe conformational variability. For example, prior HDX-MS and kinetics studies on murine iNOS identified CaM residues K21 and K30 in modulating IET rates (8). However, these residues did not form clear contacts with the heme domain in their docking model (8), suggesting their involvement may depend on conformational shifts not represented in a single structural model. Our XL MS data now detect interactions involving K30, including cross-links that exceed the distance threshold based on the top AlphaFold2 model—supporting the idea that CaM samples multiple orientations on the heme surface.

Together, the experimental evidence reinforces the view that FMN–heme and CaM–NOS interactions are dynamic and structurally heterogeneous—properties that are essential for function but not captured by a single computational structural model. The wide distance range of cross-links, in particular at the docking interfaces (Tables 1 and 2), indicate a conformation sampling mechanism in which extensive motions at the domain-domain interacting interfaces give a range of dynamic donor-acceptor complexes, only a subset of which are IET-competent (23). The partnering modules (CaM, heme, and FMN domains) form dynamic complexes (24) to balance between rapid electron transfer and efficient turnover of the enzyme.

### Effect of the E546N mutation on cross-links

We compared the relative abundances of cross-links in the PRM runs for the dimeric bands of the wt and E546N human iNOS oxyFMN samples. The mutation-induced cross-linking changes were illustrated in a volcano plot (Fig. 4) and a heatmap (Fig. S3). To improve the robustness of quantitative analysis, the PRM measurements were acquired for four independent cross-linking reactions and two technical replicates each. Notably, numerous cross-links appear at the far left of the X-axis in the volcano plot (Fig. 4), reflecting large negative fold changes corresponding to their presence in wt samples but complete absence in the E546N mutant. These were grouped into Cluster 1, represented as the blue dots with log_2_(E546N/wt) values smaller than −14. Although a fold-change cutoff of 1.5 has been commonly used to capture more subtle differences, we herein adopted a more stringent threshold of 2 to ensure robust and reliable comparative quantitation. This cutoff, combined with statistical filter (*p*-value < 0.05) and false discovery rate (FDR) controlled at 1%, minimizes false positives to reveal mutation-induced changes with potential functional significance.

Table 3 lists the cross-links with significantly decreased abundance or absence in the E546N mutant; log_2_(E546N/wt) values are provided under log_2_Fold-change. The observed decreased abundance in such a large number of cross-links reflects notable conformational changes in the mutant vs. the wt. Similar to previous sections, we focused on comparing inter-subunit FMN–heme cross-links, as these are most relevant to the formation of the docked CaM/FMN/heme complex for efficient FMN–heme IET. To reduce complexity in interpretation at this stage, we also prioritized cross-links that fall within a 40 Å distance restraint in the docked structural model, since those exceeding this distance likely reflect additional dynamic factors or alternative conformations (see below). Applying these criteria yielded three inter-subunit heme–FMN cross-links: 88–696, 155–607, and 158–607 (highlighted in Fig. 4). Notably, K607 lies near the conserved E603 at the FMN–heme docking interface; E603 and E546, two negatively charged residues on the FMN domain surface, are thought to facilitate interdomain FMN–heme docking through electrostatic interactions with positively charged residues on the heme domain (6). Our qXL MS results (Fig. 4) thus demonstrate that the E546N mutation significantly disrupts the docked state at this interface. Notably, the 88–696 cross-link is among those highlighted in Cluster 1 of Figure 4, which are absent in the mutant, indicating substantial alteration in the FMN–heme docking. This decrease likely reduces the docked-state population to the point that these cross-links become too low in abundance to be detected by LC-MS/MS. Importantly, this does not necessarily imply that docked states are completely absent in the E546N mutant—indeed, the mutant retains some IET activity (7)—but rather that docked conformers become much less populated and difficult to capture with XL MS.

**Table 3:** Cross-links with significantly decreased abundances by the E546N mutation^a^

Cross-links	log_2_Fold-change	Subunit−Subunit	Shortest distance in the top wt AlphaFold2 model (Å)
Absent in E546N mutant^b^			
88–123 heme-heme	−19.5	inter	14.36
103–113 heme-heme	−20.6	inter	8.45
103–155 heme-heme	−21.4	intra	21.27
103–158 heme-heme	−14.0	intra	23.43
88–516 heme-helix^c^	−18.8	inter	26.11
88–509 heme-helix^c^	−16.3	inter	26.09
88–696 heme–FMN	−17.4	inter	28.37
88–722 heme–FMN	−16.7	inter	47.08
103–722 heme–FMN	−14.7	inter	62.02
103–678 heme–FMN	−17.1	inter	52.23
13–88 CaM-NOS	−14.9	inter	36.42
30–88 CaM-NOS	−15.9	inter	38.48
94–88 CaM-NOS	−17.3	inter	18.57
115–88 CaM-NOS	−15.1	inter	20.34
148–103 CaM-NOS	−15.5	inter	38.47
Significantly decreased^d^			
105–689 heme–FMN	−1.86	inter	59.96
155–607 heme–FMN	−2.08	inter	20.78
158–607 heme–FMN	−1.16	inter	20.78
75–516 CaM-helix^c^	−1.04	intra	16.31

a Data were obtained from the dimeric bands of iNOS oxyFMN samples.

b These cross-links are highlighted under Cluster 1 in Figure 4. Cross-link pairs 88–123, 103–155, and 115(CaM)−88 each appear as two dots in the volcano plot due to peptide modifications, including carbamidomethylation of cysteine and methionine oxidation.

c The CaM-binding helix is located within the linker region that connects the FMN and heme domains.

d Only FMN–heme and CaM−NOS cross-links showing significantly decreased abundance (log_2_Fold-Change between −14 and −1) are listed.

In addition to the heme–FMN cross-links, Table 3 includes three other types: heme–heme, heme–CaM-binding helix, and CaM–NOS. These findings indicate that the single charge-neutralizing mutation at E546 induces extensive conformational changes across the NOS domains and CaM. For example, several CaM−heme(NOS) cross-linked pairs — 13−88, 30−88, 94−88, 115−88, and 148−103 — become absent in the E546N mutant. Furthermore, the CaM–NOS cross-link 75–516 is significantly decreased, suggesting perturbation of the interaction between CaM and the CaM-binding helix. These results support altered conformations in E546N mutant, in which CaM's engagement at its heme(NOS) docking sites (NOS residues 88 and 103) is somewhat destabilized. Importantly, CaM–NOS docking is known to facilitate FMN–heme domain-domain docking, and *vice versa* (8), underscoring the interdependence of these interactions. Taken together, our qXL MS findings demonstrate that disruption at the FMN–heme docking interface by the E546N mutation can propagate through the larger structural framework of iNOS, destabilizing CaM–NOS interactions and subsequently impacting the formation of the optimally FMN/heme/CaM docked, IET-competent complex.

### Cross-link-guided structural modeling by AlphaLink2

We utilized the aforementioned PRM-validated cross-linking data to guide AlphaLink2 (25) structural predictions of both wt and E546N mutant human iNOS oxyFMN–CaM complex. The model generation first followed standard AlphaLink2 settings, including 20 recycling iterations, 25 models, Neff set to −1 (utilizing the full multiple sequence alignment (MSA)), and no removal of MSA entries corresponding to cross-linked residues. The resulting models are ranked based on their pLDDT and predicted template modeling (pTM) scores; the top-ranked model does not necessarily give the highest cross-link satisfaction. By default, the best-scoring model undergoes a final relaxation step using OpenMM, which induces only minor conformational changes; this step is not required for downstream analysis and may be omitted.

**Figure 5:**
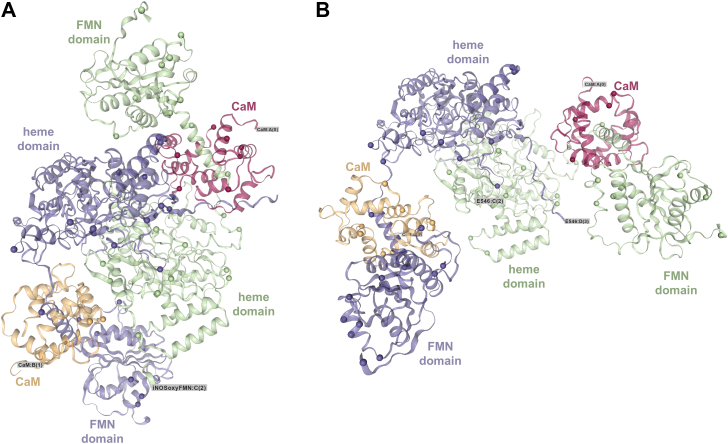
**Cross-link-guided AlphaLink2 models of iNOS oxyFMN–CaM complexes.** Top-ranked wt (*A*) and E546N mutant (*B*) models were generated using default AlphaLink2 parameters from cross-links identified by timsTOF mass spectrometry. pLDDT/pTM scores: wt, 0.868/0.869; E546N, 0.868/0.757. Images were prepared in xiVIEW with cross-linked residue pairs indicated; cross-link lines are omitted for clarity. The two structures are displayed with similar orientation in their dimeric heme domain. In wt, the FMN and heme domains are intersubunit docked (*A*), whereas the FMN domain in E546N is in an undocked, open state (*B*). The two NOS subunits are colored in *purple* and *green*, with bound CaM molecules in *orange* and *red*. CaM, calmodulin; iNOS, inducible nitric oxide synthase; oxyFMN, oxygenase/FMN; pLDDT, predicted local distance difference test; pTM, predicted template modeling; tims, trapped ion-mobility spectrometry; TOF, time-of-flight.

Interestingly, the top-ranked AlphaLink2 models of the wt and E546N mutant iNOS oxyFMN complexes adopt docked and undocked conformations, respectively (Fig. 5). In contrast, AlphaFold2 predicts nearly identical docked conformations for both proteins (Fig. S4). This divergence underscores a key limitation of AlphaFold2: it is generally insensitive to single-point mutation. AlphaFold2 was trained on protein sequence data and protein structures deposited in the Protein Data Bank before 2018. Its predictive behavior arises primarily from pattern recognition within large MSAs and learned structural correlations, rather than explicit physics-based modeling of biophysical interactions. In addition, the training data include relatively limited examples that capture the structural consequences of individual amino acid substitutions. Consequently, AlphaFold2 modeling with standard setting yields a docked conformation for the E546N mutant that is nearly indistinguishable from wt (Fig. S4).

Although the top-ranked AlphaLink2 model of the wt protein is globally similar to its top AlphaFold2 prediction (as docked states), they differ in the FMN docking geometry relative to the intersubunit heme (Fig. S5). In particular, the AlphaLink2 model places the FMN domain a bit farther away and with a distinct orientation. This observation underscores AlphaLink2’s capacity to capture alternative docking poses that reflect the dynamic nature of FMN–heme interdomain interactions in solution. In electron transfer proteins such as iNOS, the FMN domain samples multiple conformations and transiently dock at various sites to facilitate efficient electron transfer (26). This flexibility is critical for shuttling electrons across the FMN–heme interface, and thus AlphaLink2’s capacity to model these distinct docking geometries provides an advantage by revealing alternative conformations sampled by the FMN domain. The resulting models may show diversity in docking sites, relative orientations, and even conformational states (*e.g.*, docked *versus* undocked forms; see below).

Given that NOS protein natively exists in equilibrium between docked and undocked conformations, we next examined whether adjusting AlphaLink2 parameters could recapitulate some of these alternative states. Reducing the MSA depth (*e.g.*, to Neff = 42) and masking MSA information for cross-linked residues produced high-confidence models that represent distinct conformations. Tuning the MSA parameters relaxes the model’s evolutionary priors, allowing it to explore a broader range of plausible structural states. Indeed, applying these settings yielded a docked-state model for the E546N mutant (Fig. S6), whereas the default settings produced an undocked-state model (Fig. 5). Notably, both models achieved similar cross-link satisfaction scores. These experimental cross-link–guided AlphaLink2 predictions are thus both reasonable, supported by comparable confidence scores (Table S1) and cross-link satisfaction.

### Prediction of NOS conformations by AlphaFold2 subsampling

AlphaFold2 predicts the most probable structure for protein sequence(s), typically converging on one type of conformation among the top five structures. Importantly, multidomain proteins like NOSs adopt multiple functionally relevant states—such as docked and undocked conformations (Fig. 1)—critical for their activity, which standard AlphaFold2 modeling cannot capture (12, 13). To generate diverse models and explore multiple conformations, several emerging strategies have been developed in the field. These include the use of shallow MSAs, reduced recycle numbers (27, 28), and random seeds (29) to predict conformational ensembles. In silico mutagenesis using SPEACH-AF (30) has shown comparable performance depending on the specific protein context. Another approach, AF-cluster, utilizes DBSCAN to cluster MSAs and generate subsampled alignments based on the minimum cluster sizes (31, 32). Although AF-cluster can enhance conformational diversity, it may reduce model accuracy due to shallower MSA depth and has not yet been validated for multimers.

For NOS proteins, our previous AlphaFold2 subsampling runs using reduced MSA depth successfully generated an open-state model of the nNOS oxyFMN–CaM protein complex with a pTM score of 0.609 (13), where the FMN domain is positioned away from, but en route to docking on, the heme domain of the other subunit (13); the AlphaFold2 predictions, however, yielded five top-ranked structures, two of which had low pTM scores (0.468 and 0.388) and exhibited misaligned NOS domains. Although the other three models captured some conformational variability, the limited sampling resulted in a small model pool that was insufficient for meaningful clustering analysis in detail.

To address this, we expanded the AlphaFold2 subsampling approach by using seeds number of 32 to generate a larger ensemble of 160 structural models. We aimed to cluster these models to identify distinct conformational states. We then mapped cross-links to representative structures of the clusters to reduce overall cross-link distance violations and improve coverage of cross-linked residues. This approach identifies residue pairs satisfying cross-linker restraints in alternative conformational states, revealing structural dynamics missed by single-state models. Our ensemble-based framework is presented in this section.

To explore iNOS conformational diversity, the following AlphaFold2 setting was used: max-msa 128:256, num-recycle 1, use-dropout, num-seeds 32. This setup produced 160 structural models (32 seeds × 5 ranked models each). We found that enabling dropout is necessary for generating diverse structural ensembles of the iNOS protein. This setting activates dropout layers, introducing stochasticity that enables sampling of multiple plausible conformations instead of producing a single deterministic structure. To further ensure structural reliability, the resulting AlphaFold2 models were filtered based on their pTM scores, as low-scoring models (pTM < 0.5) often exhibit poor interdomain alignment or domain folding. Under these AlphaFold2 settings, all 160 iNOS models met the pTM > 0.6 threshold.

**Figure 6:**
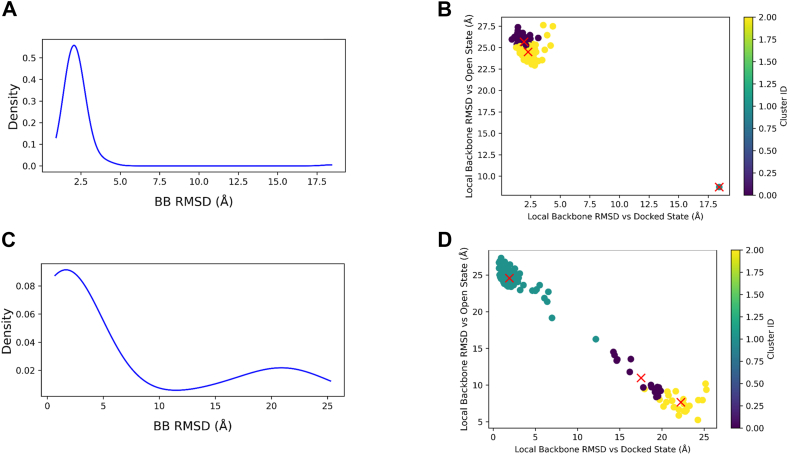
**References-guided analysis of iNOS structural ensemble relative to docked and open-state models, with local RMSD calculated for specific docking interface sites.** The docked reference structure is the top-ranked AlphaFold2 prediction using standard settings, while the open reference structure is from AlphaLink2 prediction based on the E546N cross-linking data (Fig. 5*B*). *A* and *C*, density plots of local backbone RMSD for interface residues K155, K445, R452, E546, and E603, showing their structural variation across wt (*A*) and E546N (*C*) iNOS oxyFMN ensembles relative to the docked-state reference. These conserved, charged surface residues lie at the FMN–heme docking interface. *B* and *D*, 2D RMSD clustering of wt (*B*) and E546N (*D*) ensembles. Each point corresponds to a predicted structure, plotted by its local backbone RMSD to the docked-state reference. k-means clustering with k = 3 was performed. Cluster membership is color-coded, with centroids marked by *red* “ × ” symbols, illustrating the distribution of docked-like, intermediate, and open-like states. AlphaFold2 models of iNOS were generated using max_msa=128:256, num_recycle=1, use_dropout, and num_seeds=32. From the 160 predicted structures, only those with pTM > 0.6 were retained for analysis. iNOS, inducible nitric oxide synthase; oxyFMN, oxygenase/FMN.

To quantify the conformational heterogeneity among the pTM-filtered iNOS structural predictions, we first performed a reference-based clustering analysis with MDAnalysis (33, 34). Two structurally distinct states were selected as references: the top docked-state model from AlphaFold2 (Fig. S1) and the open-state model from AlphaLink2 (Fig. 5*B*). In this approach, models are clustered according to their conformational similarity to these references, assessed by backbone RMSD to the docked and open structures as well as key intersubunit distances. This allowed us to classify models into docked-like, intermediate, and open-like subpopulations. Clustering based on local backbone RMSD of five interface residues (K155, K445, R452, E546, and E603) is shown in Figure 6; lower RMSD values indicate higher structural similarity to the docked conformation. In the wt ensemble (Fig. 6, *A* and *B*), most models cluster tightly near the docked-state reference (∼99.4%), indicating a predominantly docked conformation with minimal structural variation among the models. In contrast, the E546N mutant ensemble is much more diverse (Fig. 6, *C* and *D*), with a broad RMSD distribution and approximately 20% of the models shifted toward the open-state reference. The scatter plots also reveal a continuum of intermediate conformations distributed between the two states of E546N mutant. This broader and more rugged conformational landscape in E546N mutant supports the idea that this mutation enhances sampling of alternative, more dynamic conformations at the FMN–heme interface. MDAnalysis clustering of local backbone conformations against the open-state reference revealed comparable shifting trends (Fig. S7). Moreover, analysis of global backbone RMSD gave similar clustering results (Fig. S8). Increasing the max_msa setting to 256:512 produced a smaller, yet still notable, shift toward open-state conformations (6%) for E546N mutant relative to wt (Fig. S9), following the same overall shifting trend observed for the 128:256 setting (Fig. 6). Lower max_msa values yielded fewer models with pTM scores above 0.6 (data not shown).

The wt iNOS oxyFMN consistently produced dominant, docked conformation across different max_msa settings, as long as the max parameter was not set too low (*e.g.*, 32 or 68). In contrast, wt nNOS oxyFMN generated distinct undocked open states under the same AlphaFold2 configuration (max_msa 256:512; Fig. S10). These observations are consistent with prior studies indicating that while parameter settings can influence AlphaFold2 subsampling outcomes, the intrinsic properties of the protein sequence play a dominant role in determining structural diversity (35). As such, it is necessary to test different subsampling settings for each specific protein sequence, using metrics such as the pTM score particularly for multidomain proteins—to assess domain alignment quality and to ensure a sufficient number of high-confidence (pTM > 0.6) models for downstream clustering analysis. Generating a diverse and adequately sized ensemble of pTM-filtered models is required for capturing underlying conformational heterogeneity and enabling robust statistical interpretation of structural clusters.

**Figure 7:**
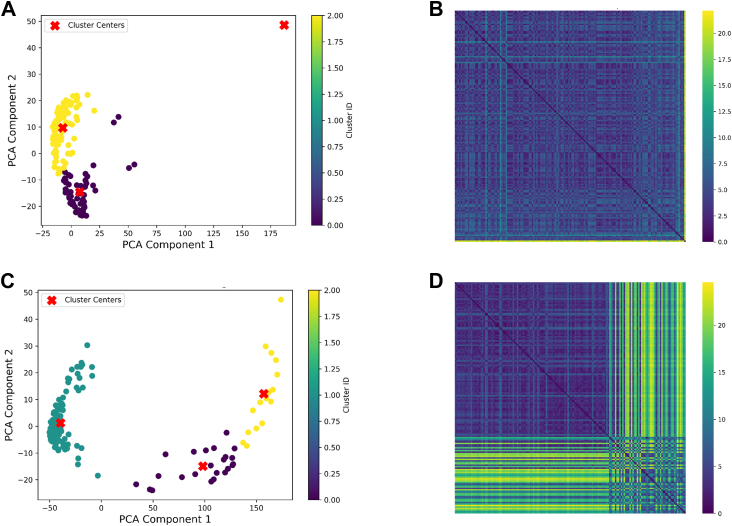
**Reference-free clustering of AlphaFold2 subsampled iNOS structural models.** Principal component analysis (PCA) scatterplot based on pairwise backbone RMSD among pTM-filtered AlphaFold2 subsampled models for wt (*A*) and E546N (*C*) human iNOS oxyFMN. Each point represents a single model projected onto the top two principal components. Colors indicate cluster membership (k-means clustering with k = 3, which partitions the data into three groups based on structural similarity), and *red* “ × ” markers denote cluster centroids in PCA space. *B* and *D*, heatmap of pairwise backbone RMSD values (in Å) among all models of wt and E546N, respectively, calculated using MDAnalysis with superposition over residues 1 to 653 of an iNOS oxyFMN chain (residues 71-723 in full-length iNOS). iNOS, inducible nitric oxide synthase; oxyFMN, oxygenase/FMN; PCA, principal component analysis; pTM, predicted template modeling.

In addition, we conducted reference-free clustering based on internal similarity metrics (Fig. 7). Reference-free clustering groups the models solely by their mutual structural differences without a predefined reference, whereas above mentioned reference-guided clustering compares the models to one or more structural states as references. Distinct block-like patterns in Figure 7 reveal conformational groupings. The principal component structures from reference-free clustering corresponds well to the representative structures derived from the reference-guided analysis for each cluster (data not shown). The broader spread of points and increased block heterogeneity for E546N mutant (panels C and D) suggest a greater conformational diversity in the mutant, compared to wt. This trend is consistent with the results of the reference-guided analysis (Fig. 6). Together, these findings indicate that, regardless of the clustering approach, the AlphaFold2-subsampling predicted E546N mutant ensemble primarily adopts three types of conformational states: one corresponding to docked configuration and two resembling the undocked, open states, with the relative population of the open states notably increased by the E546N mutation.

Notably, the principal component representing the undocked state exhibits a greater degree of opening (*i.e.*, increased separation of the FMN domain from the heme domain), compared to the undocked reference structure derived from AlphaLink2 (Fig. S11). AlphaLink2, guided by experimental cross-linking restraints, might restrain the conformational space for the FMN domain to sample. In addition, differences in their multiple sequence alignment generation—AlphaLink2 uses Jackhmmer, whereas AlphaFold2 relies on MMseqs2 — could contribute to the variation observed between their predicted models.

Importantly, the predicted trends and differences in conformational states, along with the qualitative population estimates from AlphaFold2 subsampling (Figs. 6, 7, S7, S8, S9 and S10), are generally in line with findings from multiple experimental techniques. These include pulsed EPR (7), fluorescence lifetime (36, 37), 2D IR (38), and cryo-EM (39, 40) studies, which provide complementary validation of the predicted conformational distributions in Figures 6 and 7. Pulsed EPR data demonstrated that the E546N mutant exhibits a roughly 50% reduction in the docked state population (7), consistent with the decreased docked population trend shown in Figure 6. In addition, introduction of the E546N mutation yielded 2D IR-measured dynamics that were intermediate between those for the full-length and individual oxygenase domain, consistent with a perturbation to the docked and undocked equilibrium (38). Complementing the protein spectroscopic data, cryo-EM provides spatially resolved structural snapshots that allow estimation of conformational state percentages under near-native conditions and reveal difference in population distribution between different NOS forms (40). Cryo-EM classification percentages are generally regarded as reflective of solution-state distributions of protein conformations, though sample preparation artifacts—such as protein interactions with the air-water interface—may bias observed populations. As such, caution is warranted when analyzing EM-derived distributions. For example, in one study (39), rigorous particle selection and refinement were performed, retaining approximately 5700 of 12,000 initially picked particles for final 3D reconstruction. This careful curation allowed relatively reliable estimation of structural populations. Discarding particles into “junk” classes due to damage or poorly resolved density is a routine and essential process to ensure high data quality. Their EM analyses estimated that nNOS holoprotein samples contain roughly 15% of particles in the input (docked FMN/FAD) state, 30% in the extended conformation (with the FMN domain separated from the FAD domain), and 55% in an intermediate arrangement (39). In contrast, iNOS samples showed approximately 50% input states, 30% intermediate open states, and 20% docked FMN/heme conformations (40). Taken together, the cryo-EM and pulsed EPR experimental results provide strong cross-validation for the trends predicted by AlphaFold2 subsampling computations—consistently showing a higher population of docked FMN/heme/CaM conformations in iNOS isoform relative to nNOS isoform, and a reduced docked state upon E546N mutation.

It is noteworthy and a good surprise that AlphaFold2 subsampling of the E546N mutant ensemble revealed alternative conformational states, including undocked conformations. In contrast, the standard AlphaFold2 predictions produced only docked models nearly identical to those of wt. This difference clearly demonstrates the added value of subsampling in capturing mutation-induced shifts in the conformational landscape. Although subsampling provides qualitative insights into conformational diversity, it does not provide estimates of thermodynamic stability. Because AlphaFold2 predictions are driven solely by sequence information and structural information available in the Protein Data Bank up to 2018, and lack energetic evaluation, the apparent population distributions should be interpreted with caution and validated against experimental data (see above). Further exploration using molecular dynamics simulations based on AlphaFold2-generated models and observed cross-links may help elucidate the underlying energy landscape and predict interconversion dynamics between conformational states.

We also explored additional approaches to obtain alternative conformations of homodimeric NOS systems. Generating conformational ensembles and capturing alternative structural states remain an active and evolving area of research (41, 42). Notably, this area has garnered considerable attention due to its biological and structural importance. Specifically, we evaluated emerging methods such as BioEmu (43) and AlphaFlow (44) for their applicability to NOSs. However, both tools are currently limited to monomeric proteins. In particular, the BioEmu developers explicitly state on their GitHub repository that “this code only supports sampling structures of monomers.” AlphaFlow primarily targets side chain rearrangements and small backbone motions (44) and is unlikely to capture the large-scale conformational changes observed in our study, either. Since our system involves a dimeric FMN–heme complex, these approaches are presently unsuitable for predicting its conformational ensemble. To our knowledge, AlphaFold2 with subsampling currently offers the most appropriate framework available for modeling conformations of multimeric proteins.

### Reducing overall cross-link distance violations *via* a representative structure pool

**Figure 8:**
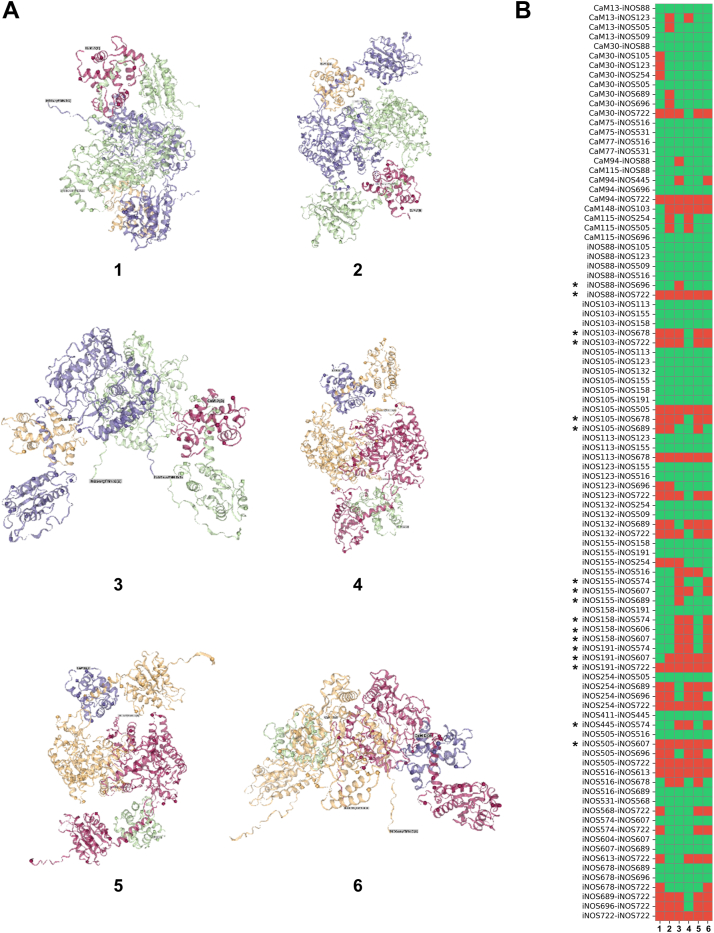
**Selected representative iNOS oxyFMN–CaM structural models and cross-link satisfaction heatmap.***A*, selected structural models of the homodimeric iNOS oxyFMN–CaM complex, representing both docked states (models 1, 2, 4, and 5) and undocked conformational states (models 3 and 6) alongside multiple orientations. *B*, heatmap showing satisfaction of all cross-links across the six models. Each row corresponds to a unique cross-linked residue pair, labeled by protein chain (iNOS or CaM) and residue number; columns represent individual structural model datasets. *Green* indicates cross-links that satisfy the 40 Å distance threshold, and red denotes those exceeding this cutoff. The inter-subunit heme–FMN cross-links in Table 1 are marked by asterisks in the Figure. iNOS, inducible nitric oxide synthase; oxyFMN, oxygenase/FMN.

We examined a pool of representative structures to assess whether conformational ensemble could reduce the cross-link distance violations (*e.g.*, in Table 1). Specifically, we selected six representative structures (Fig. 8*A*), including docked models 1 and 2, undocked model 3 from the top-ranked AlphaLink2 predictions, and three representative conformers (models 4–6) derived from MDAnalysis of AlphaFold2 subsampling. All selected models had high confidence scores and span a broad range of docked and undocked states, as well as diverse docking orientations. The pdb file of each model was imported into xiVIEW to map the wt iNOS cross-linking data (the same dataset used for Fig. 3). This process generated files that contain the three-dimensional distance for the cross-linked lysine pairs, along with their corresponding chain identifiers. Cross-link satisfaction was then binarized per pair across all six models using a 40 Å threshold. If a cross-link distance was consistent with the ensemble in at least one of the six models, we considered it satisfied in the overall structural pool. This enabled a systematic assessment of cross-link satisfaction across the ensemble.

The heatmap in Figure 8*B* illustrates the resulting pattern of satisfied and violated cross-links among the six conformational states. These visualizations highlight both conserved cross-linking patterns and variable contacts that report on dynamic structural features and intersubunit interactions. For example, the inter-subunit heme–FMN cross-links (asterisks in Fig. 8*B*) show variable satisfaction across the different cross-links: of the eight violations in Table 1 when mapped onto the top AlphaFold2 model (which adopts in a docked conformation), five become satisfied in at least one of the other models, while the other three (88–722, 191–722, and 505–607) remain consistently in violation of the distance restraint across the six models. Notably, residue 722 lies within a flexible C-terminal region (Fig. S1), which readily accounts for violations of the 88–722 and 191–722 pairs. Overall, 90% of all the experimentally observed cross-links were consistent with the distance threshold in at least one conformation, demonstrating that the conformational ensemble as a whole is broadly consistent with the cross-linking data. The remaining ∼10% of violations may reflect inherent flexibility (e.g., the C-terminus) or transient states not sampled in the models. In contrast, only 56 to 71% of cross-links satisfied the threshold in any single structural model (e.g., Fig. 3). Taken together, these findings indicate that the DSBU cross-linker captures lysine–lysine proximities sampled across multiple NOS conformational states coexisting natively in solution, underscoring the value of an ensemble-based approach, which we have applied here for the first time, rather than relying on a single structure to interpret the experimental cross-linking data.

Importantly, cross-linking violations, often previously ignored or viewed as undesirable, may actually provide more valuable insights than those within the threshold. Although threshold-compliant cross-links typically validate existing structure or structural model, violations strongly suggest alternative conformational states or dynamic transitions at specific regions. In this case, the distance violations in Tables 1 and 2 likely reflect functionally relevant domain dynamics essential for interdomain electron transfer. These data highlight the value of XL MS in capturing structural heterogeneity that single, top-ranked model alone cannot resolve. Importantly, ensemble models can help reduce such distance violations by accounting for dynamic structural heterogeneity (Fig. 8). It is important to note that cross-links represent experimental observations that capture the actual conformational states of the protein system under near-native, in-solution conditions, and structural models and conformations should be constructed and refined to satisfy these spatial restraints while explicitly accounting for the protein's inherent conformational flexibility.

In parallel with our AlphaFold2-based modeling, we explored the potential of AlphaFold3 using its publicly available web server to model structures and conformations of NOSs. Because the current version of AlphaFold3 does not support FMN as a cofactor, we used FAD as a close structural analog. The top-ranked AlphaFold3 models for both wt and E546N oxyFMN proteins were in a docked state (data not shown), being generally consistent with those generated by AlphaFold2. However, AlphaFold3 currently does not support user-defined MSA subsampling, a feature that has proven critical for capturing the conformational heterogeneity of iNOS and nNOS oxyFMN constructs (Figs. 6, 7 and S10). Given these considerations, we relied on AlphaFold2 with MSA subsampling in this work to capture the dynamic nature of the NOS oxyFMN proteins. Notably, the AlphaFold3 source code was recently released on GitHub, and as the platform continues to develop—potentially incorporating enhanced modeling flexibility and more types of cofactor—it may enable more diverse, customized applications and become a valuable tool for studying the structural heterogeneity and conformational landscape of NOS and other multi-domain, cofactor-dependent protein systems.

## Conclusions

In this study, we employed qXL MS to investigate the impact of the E546N mutation at the intersubunit FMN–heme docking interface on the structural dynamics of human iNOS oxyFMN-CaM complex. By mapping cross-links onto high-confidence AlphaFold2 models, we were able to distinguish both intersubunit and intrasubunit cross-links at domain-domain interacting sites. The qXL MS results clearly demonstrate that the charge neutralization disrupts interdomain FMN–heme docking, reducing the population of the docked states. This perturbation induces allosteric changes that propagate across the CaM–iNOS interfaces, underscoring the pivotal role of the conserved E546 residue in regulating the conformational dynamics, particularly in facilitating the FMN–heme IET.

Interestingly, cross-link-guided AlphaLink2 modeling revealed distinct conformations for the mutant compared to wt. We further showed that AlphaFold2 subsampling suggests alternative conformational states and qualitative shifts in their relative populations in the E546N mutant—features not captured by standard AlphaFold2—and that these shifting trends are generally in line with experimental data reported in the literature, including pulsed EPR, cryo-EM and 2D IR. Notably, the predicted shifting trend in relative population also aligns with our comparative qXL MS experimental results. These differences likely stem from sequence variations, such as a single hotspot mutation, which reshape the domain-docking energy landscape and bias the equilibrium toward open or docked conformations. Understanding such distinctions may help explain the unique physiological roles and regulatory responses of individual NOS isoform. Although a detailed comparison across NOS isoforms is beyond the scope of this work, our findings suggest that sequence-dependent effects may underpin isoform-specific regulation. Ongoing studies, including molecular dynamics simulations, will be the subject of future reports. These investigations will be valuable for resolving the observed distinctions and elucidating how sequence variations shape and modulate functional protein dynamics within the NOS enzyme family.

To better account for the protein’s conformational heterogeneity and the ensemble-averaged nature of XL MS, we evaluated cross-link satisfaction across an AlphaLink2 and AlphaFold2 structural ensemble. Since cross-linking captures multiple natively coexisting states, assessing distance satisfaction using a single structure alone may produce misleading distance violations. To address this, we examined the cross-links across several representative structural models that encompass both docked and undocked conformational states, and considered cross-links satisfied if they met the distance threshold in any representative model. This approach more accurately reflects conformational diversity and reduces apparent violations to 10%. Excitingly, distance violations, often overlooked or regarded as undesirable, now provide added, invaluable insights by revealing alternative conformational states and dynamic transitions. In this work, we demonstrate that XL MS reveals structural heterogeneity often missed by single structural models, with conformational ensemble satisfying experimental cross-link restraints while capturing inherent dynamic flexibility.

Collectively, our integrative qXL MS and AlphaFold2 subsampling approach provides a novel framework for mapping and interpreting mutation-induced structural dynamics at docking interface. This method not only deepens our understanding of the regulatory mechanisms of NOS but also establishes a generalizable protocol for investigating conformational plasticity and functional protein dynamics in multidomain protein systems. The ability to detect shift in structural ensembles associated with specific sequence changes or regulatory events is poised to inform and inspire broader efforts to dissect allosteric regulation and domain communication. Future investigations may further extend this framework by incorporating molecular dynamics simulations and exploring how external cues modulate the conformational and energetic landscapes.

## Experimental procedures

### Cross-linking reactions

The wt and E546N human iNOS oxyFMN proteins were overexpressed in *Escherichia coli* and purified (7). The iNOS protein has to be coexpressed with CaM due to the propensity of iNOS to aggregate when the hydrophobic residues within its CaM-binding region are solvent-exposed. The purified iNOS-CaM protein complex was buffer exchanged into HEPES buffer (20 mM HEPES, 400 mM NaCl, 10% glycerol, and 1 mM CaCl_2_, pH 7.5), and proteins with only one freeze-thaw cycle were used throughout the cross-linking reactions for better consistency. The final concentration of iNOS oxyFMN was 5 μM in reaction buffer (pH 7.5) that contains 20 mM HEPES, 100 mM NaCl, and 1 mM CaCl_2_. 0.5 μl 20 mM disuccinimidyl dibutyric urea (DSBU) in anhydrous dimethyl sulfoxide was added to a final DSBU concentration of 250 μM; the total volume was 40 μl. The cross-linking reaction was maintained for 30 min at room temperature and the reaction was quenched by adding 1 M Tris buffer (pH 7.8) to a final Tris concentration of 50 mM and incubated for 10 min.

Forty microliters of the cross-linking reaction solution was then mixed with 15 μl of 4× SDS sample buffer (Bio-Rad, Cat. # 1610747) and 6 μl of 10× reducing agent (500 mM DTT stock), and heated at 95 °C for 8 min. The denatured sample was loaded onto 4 to 15% Mini-PROTEAN TGX Precast Gel (Bio-Rad, Cat. # 4561083EDU) and electrophoresis was performed at 80 V for 2 h. After electrophoresis, the gel was transferred to a staining box with deionized water and rinsed 2 to 3 times. It was then fixed in a solution of 40% methanol, 10% acetic acid, and 50% water for 1 h at room temperature. Following fixation, protein bands were stained using water-based Coomassie Staining Solution (Bio-Rad) for 1 h or overnight at room temperature on a horizontal shaker (60 rpm). The gel was destained with water, replacing the water every 30 min, until the background was clear. Protein bands of interest were excised into ∼1 mm × 1 mm pieces using a clean scalpel, rinsing the blade between samples to prevent contamination, and stored at 4 °C for up to 1 week before in-gel digestion.

The molar ratio of DSBU to iNOS oxyFMN in the reaction buffer was optimized to minimize higher-order aggregates and over cross-linking (45, 46). An [iNOS oxyFMN]:[DSBU] ratio of 1:50 was selected as the optimal condition, as it consistently produced both monomeric and dimeric protein bands while minimizing the formation of higher-order aggregates (see Fig. S12). The broad band observed at the dimeric molecular weight is consistent with intersubunit cross-linked protein, since those without covalently cross-linked subunits would dissociate into monomers under the denaturing conditions of the gel.

As a control for our cross-linking reactions and LC-MS/MS experiments, bovine serum albumin (BSA) was cross-linked with DSBU at a 1:100 molar ratio ([BSA]:[DSBU] = 10 μM:1000 μM) in parallel with the iNOS oxyFMN cross-linking reactions; Fig. S13. BSA was selected as a control because it is a readily available, well characterized, globular, and stable protein (47). For each sample condition, at least three parallel cross-linking reactions were performed.

### In-gel digestion

To remove the Coomassie dye, the excised gel pieces were destained by washing with 25 mM ammonium bicarbonate in 50% acetonitrile. Cysteine residues were reduced by incubating the gel pieces with 10 mM DTT at 56 °C for 1 h. Alkylation was then conducted by treating the gel pieces with 54 mM iodoacetamide at room temperature in the dark for 30 min. After thorough washing with 50 mM ammonium bicarbonate (pH 8.5), the gel pieces were dehydrated with acetonitrile. Excess solvent was removed, and the samples were dried using a Speedvac. Digestion was initiated by adding MS-grade Trypsin-LysC solution (Promega) and incubating overnight at 37 °C. Peptides were extracted from the gel with 50% acetonitrile containing 5% formic acid, dried, and then reconstituted in 0.1% to 0.5% formic acid (pH 2–4). The peptide solution was transferred to low-binding vials for LC-MS/MS analysis.

### LC-MS/MS experiments on timsTOF

LC-MS/MS analysis was performed on a timsTOF fleX MALDI 2 mass spectrometer (Bruker Daltonics) equipped with a CaptiveSpray nanoelectrospray ion source (Bruker Daltonics), coupled to a Dionex Ultimate 3000 RSLCnano System (Thermo Fisher Scientific). Peptides were eluted from an Acclaim PepMap 100 trap column (75 micron ID x 2 cm, Thermo Fisher Scientific) after a 5-min wash with a solvent mixture of 2% acetonitrile, 98% water and 0.1% formic acid at 5 μl/min, onto a PepSep analytical column (25 cm × 75 μm, C18, 1.5 μm; Bruker Daltonics) maintained at 50 °C. The mobile phases consisted of 0.1% formic acid in water (A) and 0.1% formic acid in acetonitrile (B), delivered at an analytical flow rate of 300 nl/min, with a gradient of: 2% B for 8 min, 2 to 40% B from 8 to 54 min, 40 to 85% B from 54 to 56 min, hold at 85% B from 56 to 60 min, 85-2% B from 60 to 62 min, and re-equilibrate at 2% B from 62 to 70 min.

DDA-PASEF was conducted with a source voltage of 1600 V. The MS and MS/MS scan range was set to 375 − 1700 m/z, and the 1/*K*_0_ range was 0.60 to 1.60 V·s/cm^2^ over a ramp time of 200 ms. Ten PASEF MS/MS scans were acquired per cycle with a 200 ms accumulation time. An active exclusion time of 0.5 min was applied to precursors that reached an intensity of 100,000. Each sample was analyzed in two separate runs: one with MS/MS stepping to enable two collision energy ramps as functions of ion mobility (Ramp 1: 23 eV for 1/*K*_0_ = 0.73 V·s /cm^2^ and 59 eV for 1/*K*_0_ = 1.6 V·s/cm^2^; Ramp 2: 23 eV for 1/*K*_0_ = 0.73 V·s/cm^2^ and 95 eV for 1/*K*_0_ = 1.6 V·s/cm^2^), and the other with a single collisional energy ramp (23 eV for 1/*K*_0_ = 0.73 V·s/cm^2^ and 75 eV for 1/*K*_0_ = 1.6 V·s/cm^2^). The MS data from the two runs were analyzed separately using MeroX (v2.0) (45), and the identified cross-links were then combined per sample.

For PRM-PASEF acquisition, a target list including all the PRM-validated (see below) cross-linked peptides of the wt and E546N mutant human iNOS oxyFMN proteins was imported, and the tims ramp and accumulation time was reduced to 100 ms. MS/MS stepping was also applied to enable two collision energy ramps as functions of ion mobility (Ramp 1: 23 eV for 1/*K*_0_ = 0.73 V·s/cm^2^ and 59 eV for 1/*K*_0_ = 1.6 V·s/cm^2^; Ramp 2: 23 eV for 1/*K*_0_ = 0.73 V·s/cm^2^ and 95 eV for 1/*K*_0_ = 1.6 V·s/cm^2^). The other settings were kept the same as DDA-PASEF. Technical duplicates for each sample were acquired in the PRM-PASEF mode.

### DDA-PASEF data analysis

The analysis of the DDA-PASEF data was performed following the procedure in the literature (48). The raw data files were first processed using Compass DataAnalysis software (v6.1, Bruker Daltonics) with a customized method (.m) script. The .mgf output files were analyzed by MeroX (v2.0) (45) to search for peptide pairs cross-linked by DSBU. For the search, only lysine (K) residues were considered as cross-linking sites, and only the pairs that contain the MS-cleavable fragments of DSBU modifications (+85.05276381 Da and +111.03202835 Da) at both sites in the MS/MS were considered. The precursor mass tolerance was set to 10 ppm and fragment mass tolerance was set to 15 ppm. The precursor mass range was set to 300 − 8000 Da. The cutoff score was first set to a highly tolerable value of 15. The generated .zhrm files were combined in MeroX (v2.0) (45), and the cross-linked peptide pairs were then filtered by the cutoff score associated with 1% FDR. As FDR for single protein analysis provides rather limited accuracy due to a small search space, cross-linked peptide hits were further examined and filtered in Skyline (49). Specifically, a csv file for the combined data was exported from MeroX (v2.0) (45) and processed with a customized R script to output a txt file containing 1/*K*_0_ information to build an ion mobility library in Skyline (v23.1). To build a peptide spectral library in Skyline, a proXL .xml file was generated for each run using the “merox2ProxlXML.jar” Java executable file, and all the resulting .xml files were imported into Skyline. After importing all .d raw files, the library peptides were examined, and those with only one library match spectrum across all reaction and technical replicates were likely to be false positive and thus were removed. An isolation list was then exported from Skyline and imported to the timsControl (v6.0, Bruker Daltonics) data acquisition software for PRM-PASEF acquisition. All information regarding the above-mentioned customized scripts and Java executable file can be found in reference (48).

Targeted MS/MS analysis by PRM significantly improves the identification and quantitation of the cross-linked peptides, which are typically of low-abundance in the protein digestion mixture. Unique cross-links with FDR < 1% in at least one DDA run were compiled into a PRM inclusion list for subsequent targeted MS experiments. This step is essential due to the typically low abundance of cross-linked peptides and rather limited reproducibility of XL MS, particularly with DDA-based quantitative XL MS. To improve sensitivity and specificity, we employed PRM, analyzing top 10 fragment ion transitions per MS1 peak. This approach improves confidence in both identification and quantitation of each cross-link. The resulting PRM peaks were symmetric with sufficient data points for quantitation. Representative examples of two cross-links are shown in Fig. S14, confirming that the PRM acquisition parameters and LC conditions were well-optimized for accurate detection of the targeted cross-linked peptides. We only included such “PRM-validated” cross-links for subsequent qXL MS data analysis.

In our experiments, we identified 96 and 114 unique cross-links in two independent batches of BSA control samples processed on separate days (Fig. S15). A previous study using a similar timsTOF equipment reported 405 unique cross-links for a 125-fold DSBU excess sample (50); those results were derived from in-solution digests of both BSA monomers and higher-order oligomers such as dimers, and their data analysis included cross-links involving serine, threonine, tyrosine, and lysine (STYK) residues. When we included STYK sites in our analysis, we identified 232 unique cross-links. Giving that we performed in-gel digestion and focused exclusively on the monomeric band, our BSA results align reasonably with the literature (50). Overall, our BSA control results demonstrate the effectiveness of the cross-linking reaction conditions, in-gel digestion, LC-MS/MS acquisition using timsTOF, and XL MS data analysis using MeroX (v2.0) (45).

Notably, we chose to include only lysine (K) cross-linking sites in our search, since analysis of timsTOF MS data for the iNOS proteins revealed that inclusion of STY residues increased the number of identified unique cross-links by more than 100% compared to lysine-only results. This outcome undermines the reaction specificity of DSBU cross-linking chemistry, which is primarily selective for lysines (51). In contrast, our XL MS data, acquired on a QE Classic Orbitrap and analyzed by the XlinkX node in Proteome Discoverer 2.5, showed that only ∼10% of identified cross-links involved STY residues (13), a result that aligns with the expected cross-linking pattern and supports the selective behavior of the DSBU chemistry (51) when STYK residues are included. The difference between the timsTOF and Orbitrap results reinforces the rationale for our choice to focus exclusively on lysine residues for timsTOF data analysis.

Of note is that the timsTOF mass spectrometry identified significantly more lysine only cross-links in iNOS oxyFMN proteins (77–96 cross-links) compared to the Orbitrap Classic system (30–40 cross-links). This enhanced performance is likely due to the combination of tims and PASEF, which improves peptide precursor separation, sequencing speed and ion utilization efficiency—particularly beneficial for low abundance cross-linked peptide mixtures. Therefore, we focus on presenting the timsTOF data in this work.

### PRM-PASEF data analysis

The .d raw data files were imported into the Skyline project that was generated from the aforementioned DDA-PASEF data analysis. The spectrum quality and peak area integration of each library peptide were examined manually. Those with S/N ratios < 3 were removed. The peak area intensities of cross-linked peptides were exported and normalized based on the summed peptide intensity of iNOS oxyFMN−CaM complex in each sample obtained from the DDA-PASEF experiment. The replicate data were averaged to calculate the abundance ratios of cross-linked peptides between the E546N mutant and wt iNOS oxyFMN. A two-sample unpaired two-tailed *t* test was performed and the corresponding volcano plot was generated using Python and SciPy library to identify cross-linked peptides with a significant abundance change (fold change ≥ 2) and *p*-value < 0.05.

### Predication of the iNOS oxyFMN structures by AlphaFold2

ColabFold v1.5.5 (52) was used to predict the wt and E546N human iNOS oxyFMN structures (standard setting: max_seq = 508, max_extra_seq = 2048). Sequence alignments were generated by MMseqs2, and the templates were detected in PDB100. The top five models generated from the AlphaFold Multimer calculations generally converged to similar structures, exhibiting docked CaM/FMN/heme architecture in an intersubunit FMN-heme docking arrangement for both the wt and E546N mutant proteins. The AlphaFold2 models were benchmarked against existing crystal structures of the NOS domains.

### Predication of the iNOS oxyFMN conformations by AlphaFold2 subsampling

To diversify the models and generate multiple conformations, a shallow number of sequences (MSA) and/or reduced recycle numbers were employed to predict conformational ensembles of several other proteins (27, 28, 29). In this work, we explored the AlphaFold2 subsampling methods to generate alternative conformations for wt and E546N mutant iNOS oxyFMN proteins. We ran AlphaFold2 with 32 seeds to generate 160 models for population and clustering analysis, following the procedure in reference (35). Various subsampling methods were tested for iNOS, including dropout and varying the number of sequence clusters. The resulting models were filtered by their pTM scores, retaining only those with scores > 0.6. These pTM-filtered models were then input as trajectories into MDAnalysis (33, 34), and RMSD was calculated relative to reference structures that include both the docked structures from AlphaFold2 and the open, undocked structural models from AlphaLink2. Reference-free clustering analysis was also conducted to complement the predictions of representative structures and provide qualitative estimates of cluster populations.

### Cross-link guided predication of iNOS oxyFMN structures by AlphaLink2

AlphaLink2 (25), an integrative modeling tool that combines XL MS data with UniFold-based (53) predictions, was installed on a local workstation and used to model the iNOS oxyFMN−CaM complex. PRM-validated cross-links were exported from xiVIEW (54), using a stoichiometry of 2 for selected proteins (i.e., dimeric iNOS oxyFMN and its bound CaM in this case) to reflect the dimeric state of iNOS oxyFMN and its bound CaM molecules. The xiVIEW export for the AlphaLink2 option includes all the cross-links with their residues referenced to the input protein sequences. Due to the homodimeric nature of the iNOS-CaM complex, the number of cross-links listed in the exported txt file was four times of the actual number of experimental cross-links. This file was converted into Excel format for further processing. Intrachain cross-links were removed using a custom Python script to reduce computational complexity and eliminate restraints primarily reflecting intradomain folding, which tends to be relatively rigid and thus not the priority of structural modeling. This intrachain filtering step allowed the AlphaLink2 analysis to focus on interchain and interdomain interactions that are more relevant to conformational variability and function. To allow for greater flexibility in modeling, the FDR score was relaxed from 0.05 to 0.20. The curated cross-link input file along with the xiVIEW-exported iNOS and CaM protein sequences were then provided to AlphaLink2. Standard AlphaLink2 modeling parameters were used as follows: the model file *AlphaLink-Multimer_SDA_v3.pt* (dated May 1, 2020) was employed, with template structures limited to those deposited prior to this date; recycling iterations were set to 20; 25 structural samples were generated; effective number of sequences (Neff) to −1; and MSAs were removed for residues involved in cross-linking by setting their values to −1. In addition to these standard AlphaLink2 runs, customizable parameters were explored to further diversify the modeling outcomes. Tailored parameters included the number of recycling iterations, number of samples, Neff for subsampling MSAs, and toggling MSA removal for cross-linked residues. These options are different ways to introduce conformational diversity, enhance sampling, or adjust relative weighting during modeling.

All AlphaFold2 and AlphaLink2 calculations were performed on a workstation running Ubuntu OS, equipped with a 48GB NVIDIA RTX A600 GPU.

## Data availability

The mass spectrometry data have been deposited to the ProteomeXchange Consortium *via* the PRIDE (55) partner repository with the dataset identifier PXD064795. The top AlphaFold2 models of wt and E546N mutant human iNOS oxyFMN proteins have been deposited to ModelArchive (56) (project ID ma-v90ny and ma-vk4y8, respectively).

## Supporting information

This article contains supporting information. (58, 59, 60, 61, 62)

## Conflict of interest

The authors declare that they have no conflicts of interest with the contents of this article.
