# Supplementary material for: Deciphering mutational effects on inducible NO synthase conformational dynamics via quantitative cross-linking mass spectrometry and AlphaFold2 subsampling
Source: J Biol Chem. 2025 Oct 27;301(11):110673. doi: 10.1016/j.jbc.2025.110673 (PMC12597282; doi:10.1016/j.jbc.2025.110673)
Supplement: Supporting Figures and Table [file mmc1.pdf]

Supporting Information

Deciphering mutational effects on inducible NO synthase conformational dynamics via quantitative cross-linking mass spectrometry and AlphaFold2 subsampling

Ting Jiang [1], Haikun Zhang [1], Gabriel Monteiro Da Silva [2], Yadav Prasad Gyawali [1], Changjian Feng [1,3] *

[1] Department of Pharmaceutical Sciences, College of Pharmacy, University of New Mexico, Albuquerque, NM 87131
[2] Department of Molecular and Cellular Biology and Biochemistry, Brown University, Providence, RI 02912
[3] Department of Chemistry and Chemical Biology, University of New Mexico, Albuquerque, NM 87131

Supplementary Figure Legends and Table

Figure S1. AlphaFold2 model confidence and predicted aligned error analysis of the wild-type (wt) human iNOS oxyFMN–CaM complex.

Figure S2. Structural alignment of top AlphaFold2-predicted iNOS domains with corresponding crystal structures.

Figure S3. Hierarchical clustering heatmap comparing cross-link abundance changes between wt and E546N human iNOS oxyFMN samples.

Figure S4. Structural comparison of AlphaFold2 models for wt and E546N iNOS oxyFMN.

Figure S5. Pairwise structural alignment of the top AlphaFold2 (brown) and AlphaLink2 (blue) models of wt iNOS oxyFMN-CaM complex.

Figure S6. Top-ranked AlphaLink2 model of the E546N human iNOS oxyFMN–CaM complex using tailored AlphaLink2 parameters.

Figure S7. Reference-guided analysis of iNOS structural ensembles using local RMSD of interface residues relative to the open-state structure.

Figure S8. Reference-guided analysis of the iNOS structural ensembles, with global RMSD calculated across the entire iNOS chain.

Figure S9. Local backbone RMSD distributions and clustering analysis of AlphaFold2 ensembles for wt and E546N iNOS oxyFMN, generated with an increased max_msa setting (256:512).

Figure S10. Local backbone RMSD distributions and clustering of AlphaFold2 ensembles for wt nNOS oxyFMN.

Figure S11. Alignment of the top AlphaLink2 undocked model and the principal component of the undocked-state cluster from MDAnalysis of AlphaFold2 subsampling ensembles.

Figure S12. SDS-PAGE analysis of DSBU cross-linking reactions of iNOS oxyFMN.

Figure S13. SDS-PAGE analysis of DSBU cross-linking reactions with BSA.

Figure S14. Representative PRM chromatograms of intersubunit NOS–NOS cross-links in wt and E546N iNOS oxyFMN.

Figure S15. Reproducibility and structural analysis of DSBU cross-links in BSA by timsTOF mass spectrometry.

Table S1. Confidence scores for top-ranked AlphaLink2 and AlphaFold 2 structural models of iNOS oxyFMN proteins

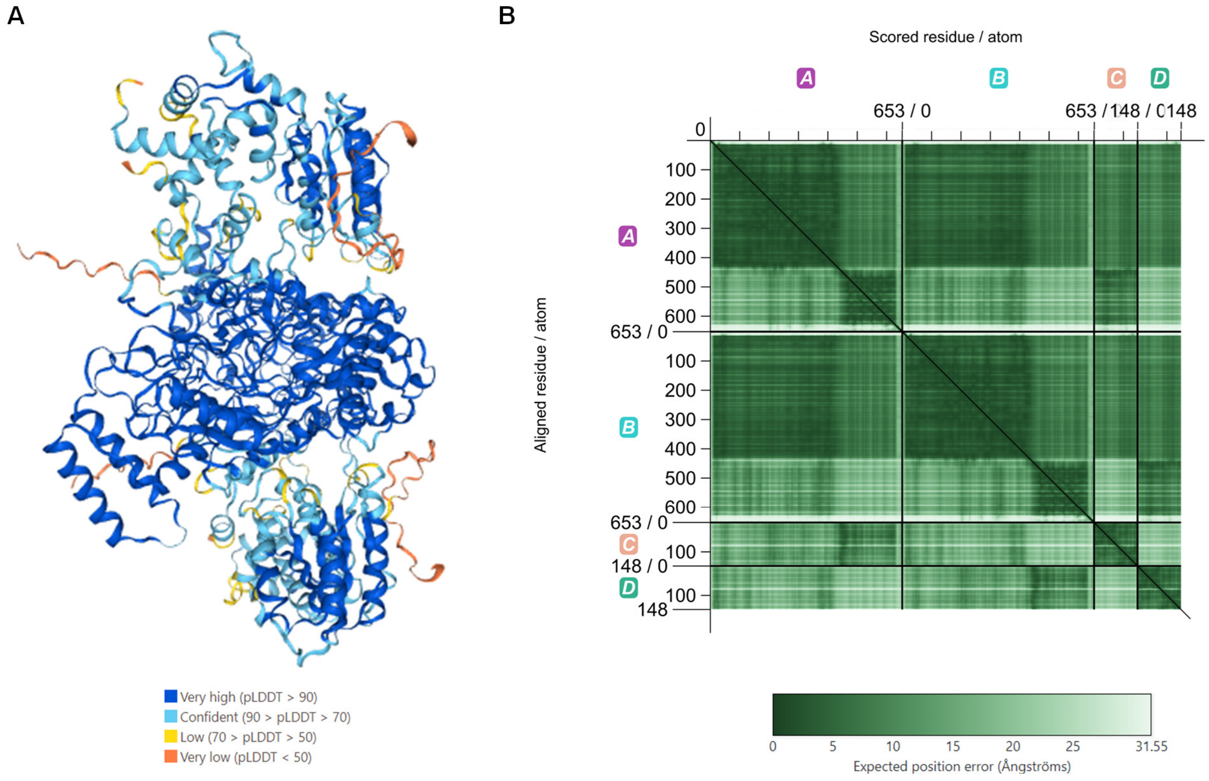

Figure S1. AlphaFold2 model confidence and predicted aligned error analysis of the wild-type (wt) human iNOS oxyFMN–CaM complex. (A) Per-residue confidence scores (pLDDT) mapped onto the AlphaFold2 top-ranked model of the wt human iNOS oxyFMN–CaM complex, with residues colored by confidence level. Model confidence metrics: mean pLDDT 87.56; pTM 0.87; ipTM 0.86. The N- and C-terminal regions (residues 71–84 and 679–723, respectively) exhibit lower confidence. (B) Predicted aligned error (PAE) plot for the same model, illustrating relative positioning confidence between domains. Diagonal blocks corresponding to individual chains (A and B for the homodimeric NOS subunits; C and D for the two bound CaM molecules) show uniformly low PAE values, indicating high confidence in internal domain structures. Off-diagonal regions, representing interdomain and intersubunit relationships, display moderately increased PAE values. Notably, the A–B and C–D blocks retain structured features, indicating moderate confidence in relative orientations between NOS subunits and CaM molecules, respectively. Higher PAE values in the A–C, A–D, B–C, and B–D blocks suggest lower confidence in NOS–CaM domain interactions, reflecting the intrinsic flexibility and multiple possible docking arrangements at this interface. These PAE patterns align with the known conformational plasticity of multidomain proteins like NOS, where flexible linkers and dynamic interfaces (e.g., FMN–heme and CaM–NOS) permit significant structural variability. Panels (A) and (B) were generated using the PAE viewer (1) with the AlphaFold2 predicted structure and associated JSON file.

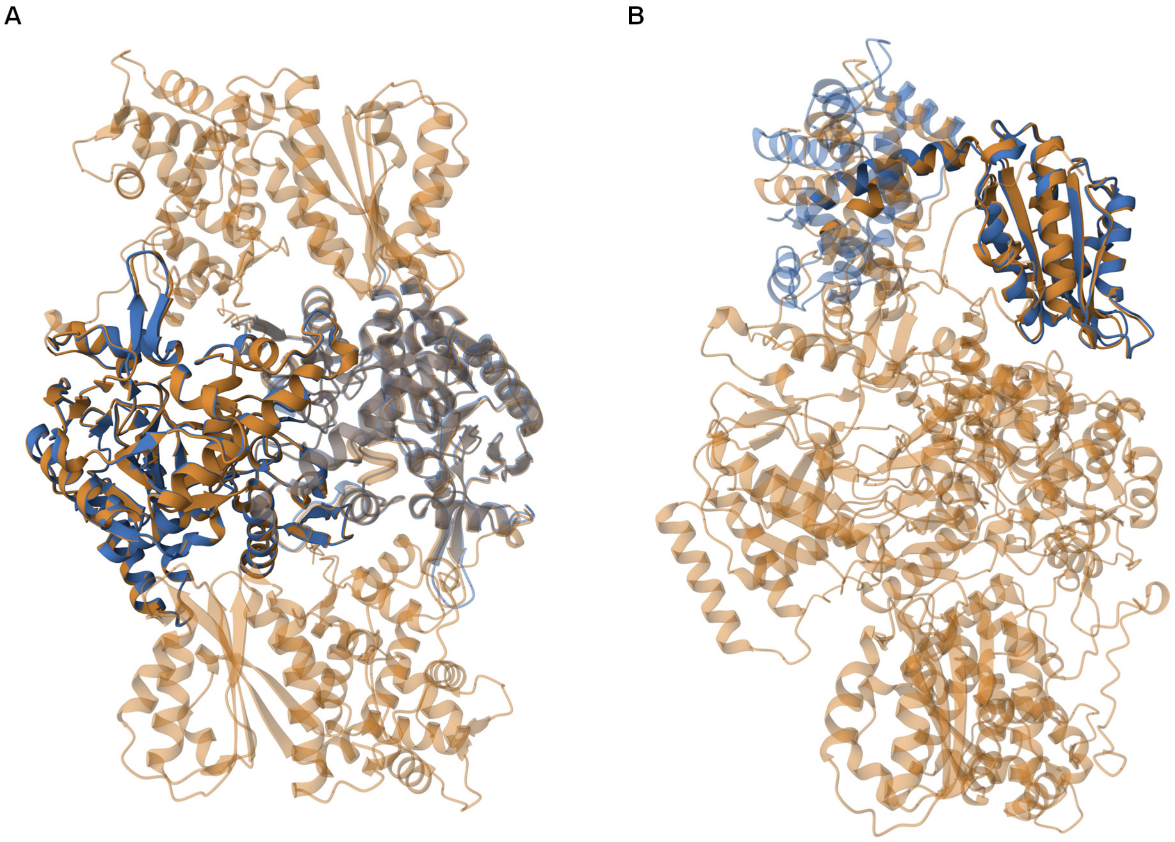

Figure S2. Structural alignment of top AlphaFold2-predicted iNOS domains with corresponding crystal structures. (A) Structural alignment of the heme domain from the AlphaFold2 model (light brown) with the human iNOS heme domain crystal structure (blue; PDB ID: 4NOS), showing an RMSD of 0.333 Å and a TM-score of 1. (B) Structural alignment of the FMN domain and bound calmodulin (CaM) from the AlphaFold2 model (light brown) with the human iNOS FMN domain–CaM crystal structure (blue; PDB ID: 3HR4), showing an RMSD of 1.25 Å and a TM-score of 0.80. Panels (A) and (B) together demonstrate that AlphaFold2-predicted domain structures closely recapitulate experimentally determined crystal structures. Structural alignments were performed using the RCSB PDB research-focused web portal (2).

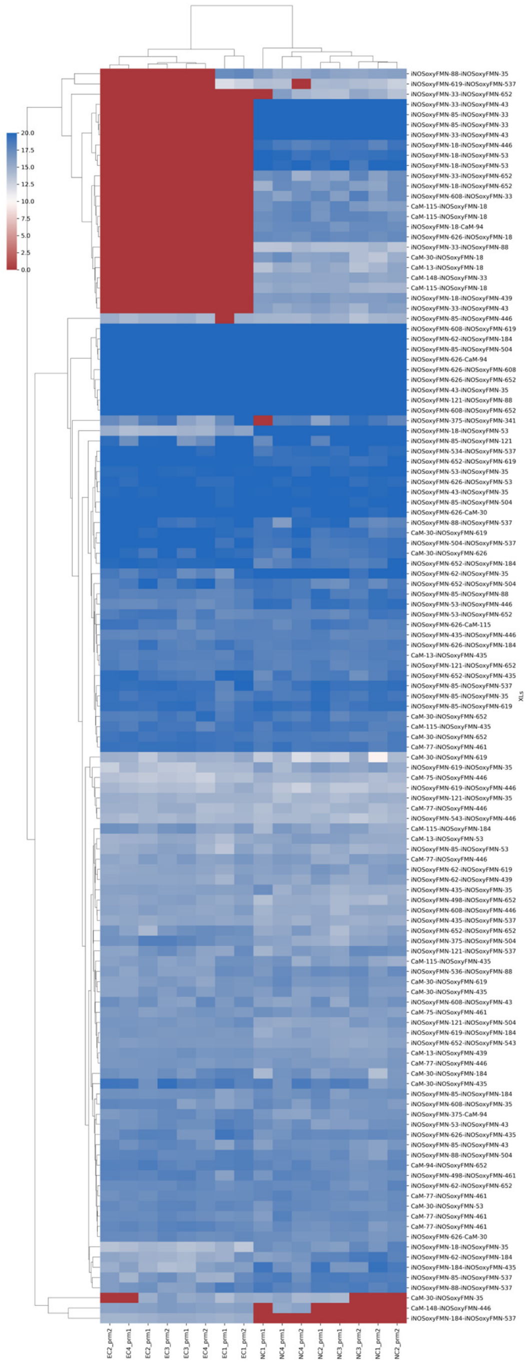

Figure S3. Hierarchical clustering heatmap comparing cross-link abundance changes between wt and E546N human iNOS oxyFMN samples. The color scale represents log_2-transformed abundance, with darker red indicating diminished cross-link abundance. Each column represents one of eight replicates per condition, consisting of four independent cross-linking reaction replicates, each measured in duplicate (technical replicates PRM 1 and PRM 2); NC denotes the wt NOS-CaM complex, and EC denotes the E546N-CaM complex. The heatmap highlights dramatic decreases in cross-links within Cluster 1 of main text Figure 4, showing consistent loss (darkest red) across all replicates. The heatmap was generated using the Python Seaborn library.

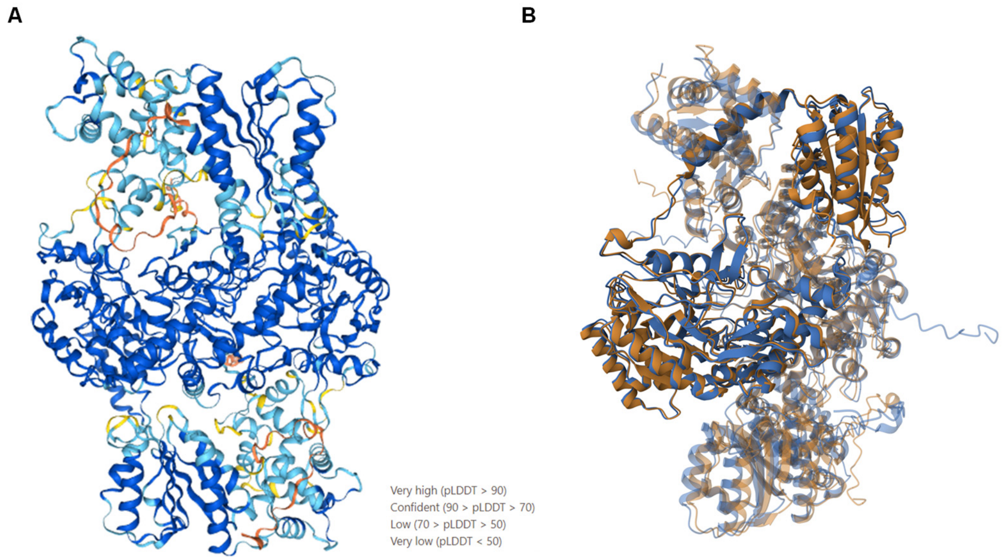

Figure S4. Structural comparison of AlphaFold2 models for wt and E546N iNOS oxyFMN. (A) Top-ranked AlphaFold2 model of the E546N human iNOS oxyFMN–CaM complex. Model confidence metrics: mean pLDDT 87.68; pTM 0.88; ipTM 0.86. (B) Pairwise structural alignment of the top AlphaFold2 models for wt (brown) and E546N mutant (blue) iNOS oxyFMN. The backbone RMSD is 1.65 Å, with a TM-score of 0.95, indicating high structural similarity.

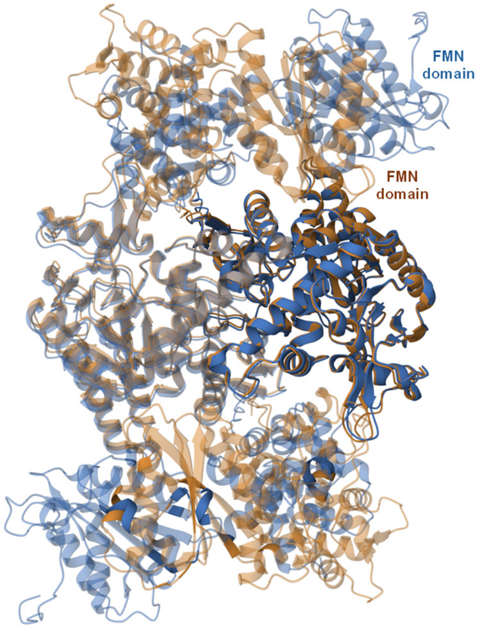

Figure S5. Pairwise structural alignment of the top AlphaFold2 (brown) and AlphaLink2 (blue) models of wt iNOS oxyFMN-CaM complex. The backbone RMSD of 3.29 Å and TM-score of 0.75 indicate moderate structural divergence. The models differ in FMN domain docking, with the AlphaLink2 model placing it farther and in a distinct orientation relative to the inter-subunit heme domain compared to the AlphaFold2 model.

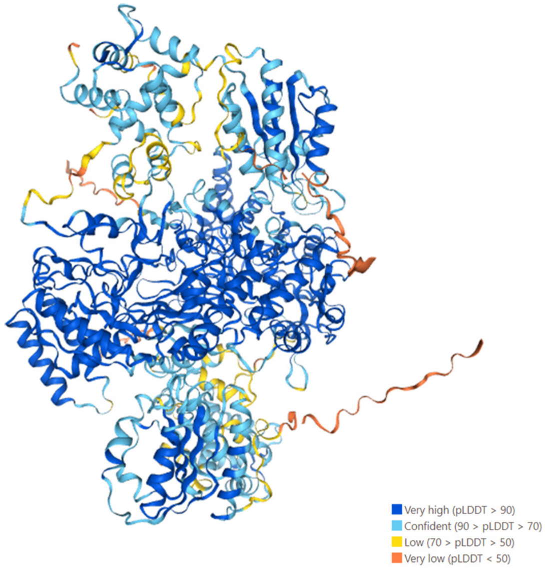

Figure S6. Top-ranked AlphaLink2 model of the E546N human iNOS oxyFMN−CaM complex using tailored AlphaLink2 parameters. Customized AlphaLink2 parameters are: recycling iterations = 3, number of samples = 25, effective MSA depth (Neff) = 42, and removal of MSA for cross-linked residues. Model confidence scores are: mean pLDDT = 87.48, pTM = 0.877, and ipTM = 0.849.

A

B

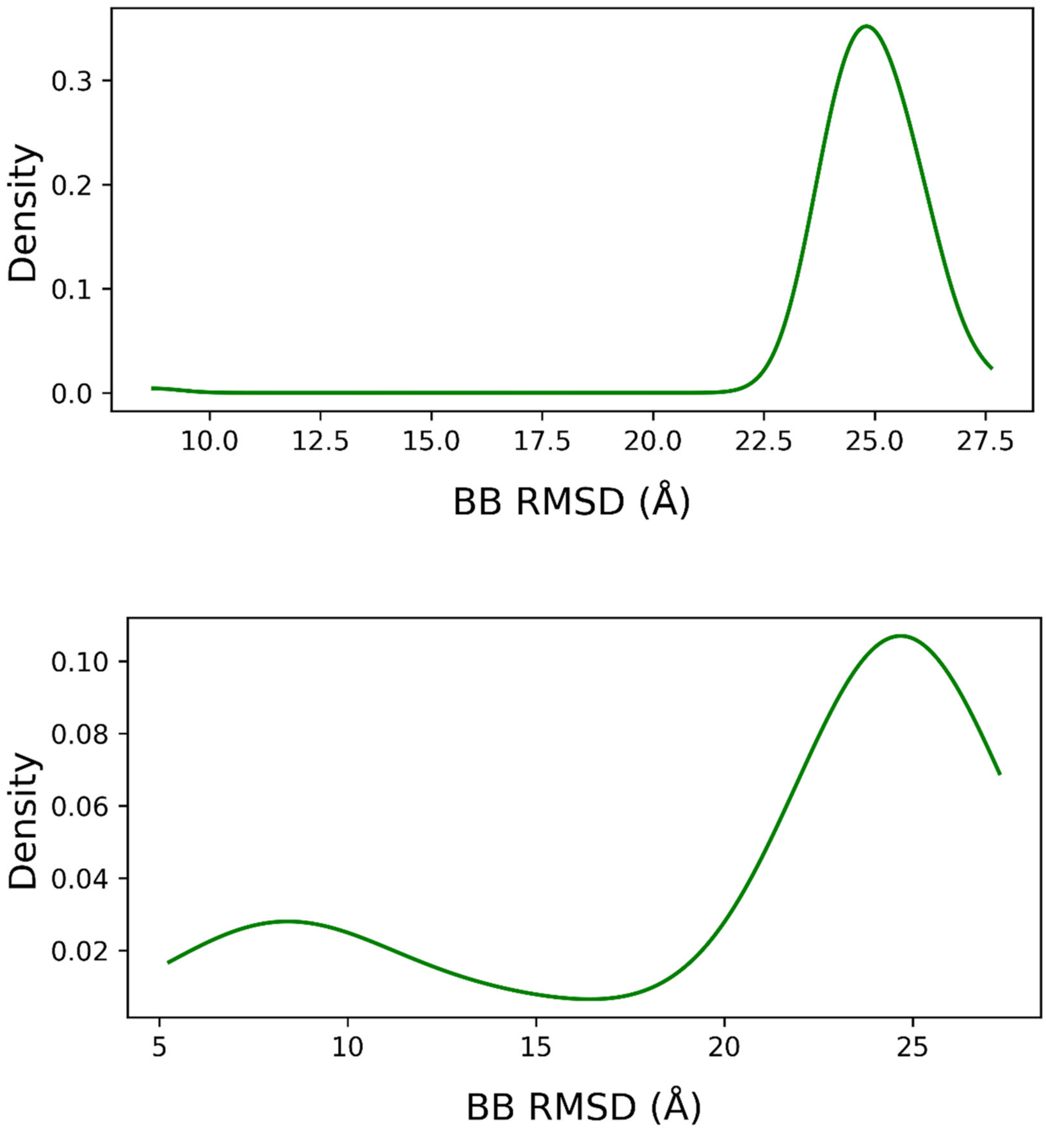

Figure S7. Reference-guided analysis of iNOS structural ensembles using local RMSD of interface residues relative to the open-state structure. Density plots of local backbone RMSD for interface residues K155, K445, R452, E546, and E603, showing their structural variation across wt (**A**) and E546N (**B**) iNOS oxyFMN ensembles relative to the open-state reference. The AlphaFold2 structures were generated using the following setting: max-msa 128:256, num-recycle 1, use-dropout, num-seeds 32. Of the 160 resulting structures, only those with pTM > 0.6 were retained and analyzed.

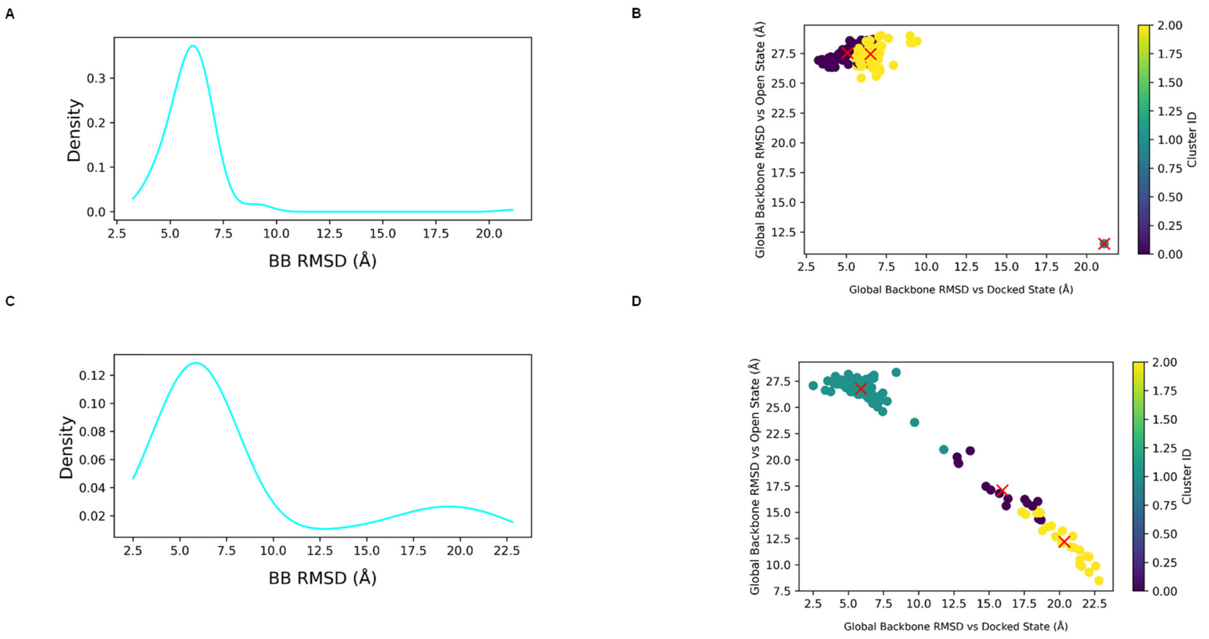

Figure S8. Reference-guided analysis of the iNOS structural ensembles, with global RMSD calculated across the entire iNOS chain. The references are the same as Figure 6 in main text. (**A, C**) Backbone RMSD density plots for the NOS chain in wt (A) and E546N mutant (C) iNOS oxyFMN ensembles, shown relative to the docked state reference. (**B, D**) Two-dimensional backbone RMSD plots illustrating conformational clustering in the wt (B) and E546N (D) ensembles. Clusters are color-coded, with red "×" symbols indicating cluster centroids.

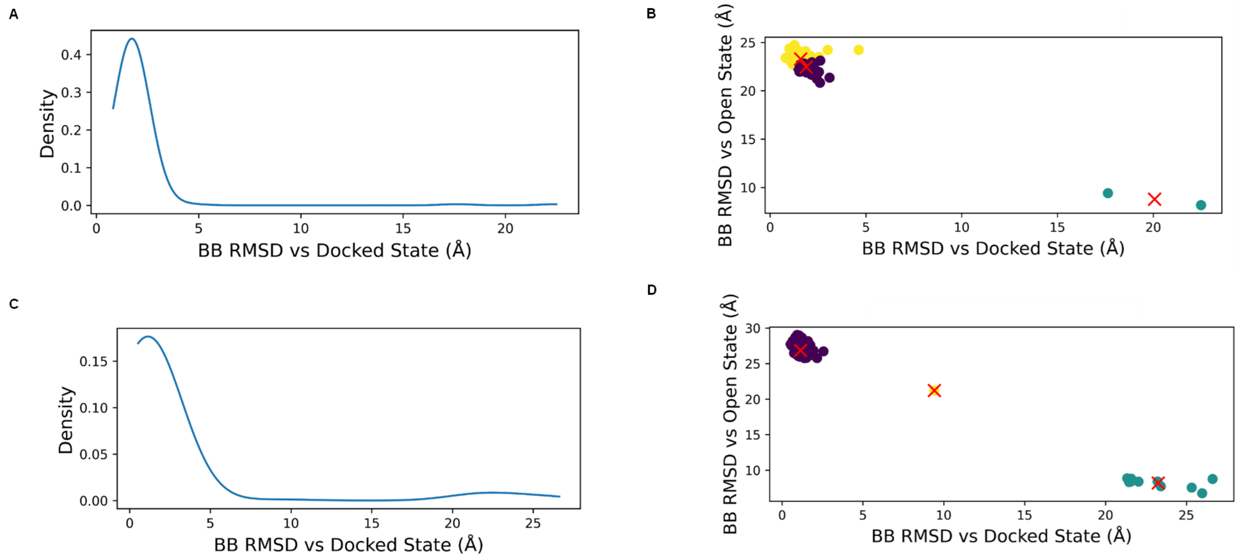

Figure S9. Local backbone RMSD distributions and clustering analysis of AlphaFold2 ensembles for wt and E546N iNOS oxyFMN, generated with an increased max_msa setting (256:512). (**A, C**) Distributions of local backbone RMSD values relative to the docked-state reference structure for ensembles of wt (A) and E546N mutant (C) human iNOS oxyFMN. The AlphaFold2 subsampling settings are: max_msa 256:512, num-recycle 1, use-dropout, num-seeds 32. Each ensemble contains 160 pTM-filtered structural models, and RMSD frequencies were smoothed using kernel density estimation. (**B, D**) KMeans clustering (k = 3) applied to the models based on their local backbone RMSD to the docked (X-axis) and open (Y-axis) reference conformations for wt (B) and E546N (D). Each dot corresponds to a single model, with colors indicating cluster membership. Red "×" symbols denote the centroids of each cluster.

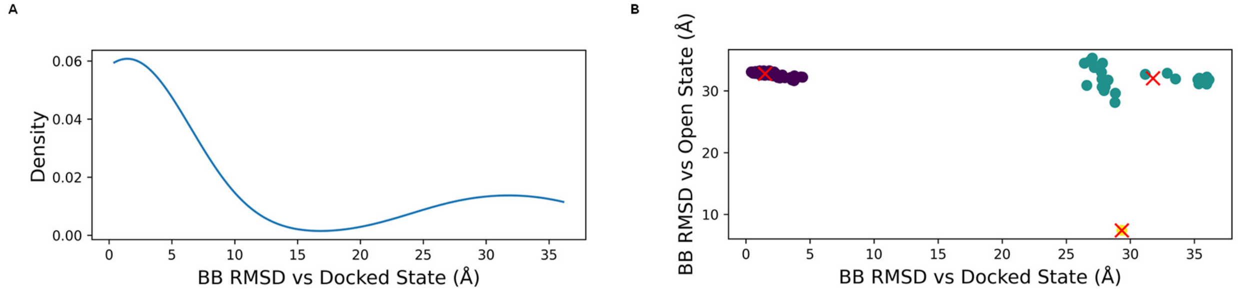

Figure S10. Local backbone RMSD distributions and clustering of AlphaFold2 ensembles for wt nNOS oxyFMN. (A) Local backbone RMSD distributions relative to the docked-state reference for AlphaFold2 ensembles of wt rat nNOS oxyFMN using settings: max_msa 256:512, 1 recycle, dropout, 32 seeds. RMSD frequencies were smoothed by kernel density estimation. (B) KMeans clustering (k = 3) of models based on RMSD to docked (X-axis) and open (Y-axis) states; centroids are marked by red "×". The wt nNOS oxyFMN exhibits greater conformational diversity than wt iNOS oxyFMN under the same AlphaFold2 subsampling settings (Figure S8).

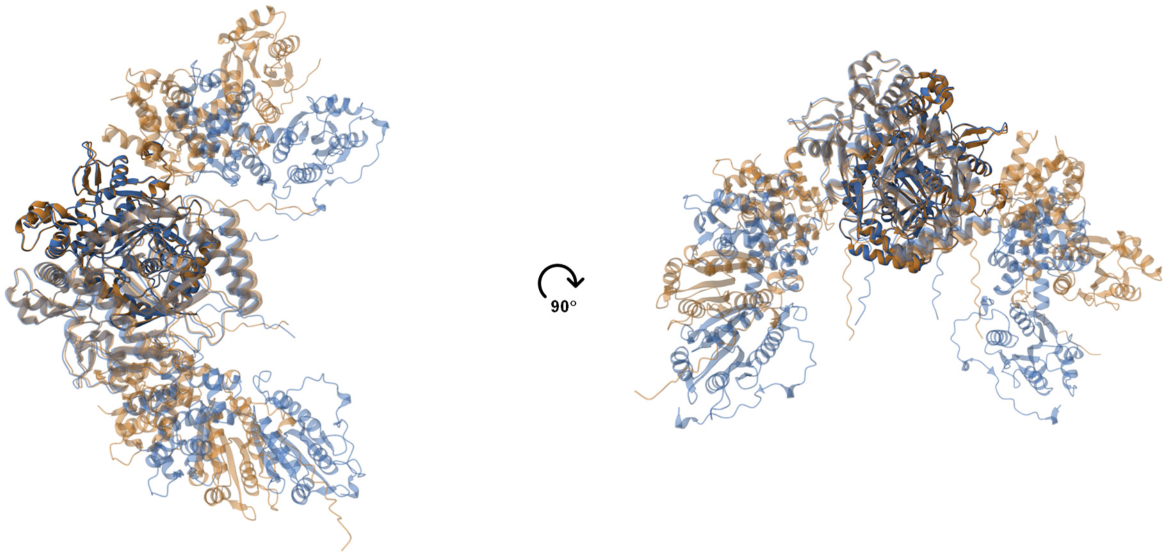

Figure S11. Alignment of the top AlphaLink2 undocked model and the principal component of the undocked-state cluster from MDAnalysis of AlphaFold2 subsampling ensembles. The backbone RMSD is 2.67 Å and TM-score 0.67, indicating moderate structural divergence.

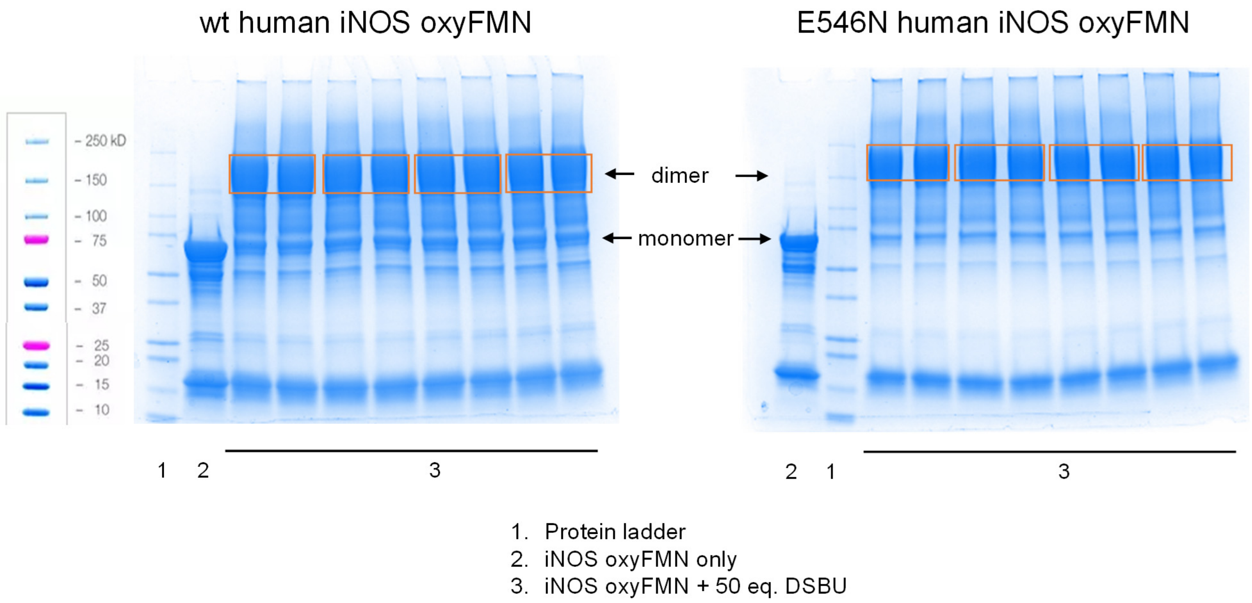

Figure S12. SDS-PAGE analysis of DSBU cross-linking reactions of iNOS oxyFMN. Protein samples were analyzed under denaturing conditions following cross-linking with DSBU. Lane 1: molecular weight marker; Lane 2: iNOS oxyFMN only; Lanes 3a–3c: replicate cross-linking reactions at a 1:50 molar ratio of protein to DSBU, each showing both monomeric and dimeric bands. Dimeric cross-linked bands (highlighted by red boxes) were excised for in-gel digestion and subsequent MS analysis. Bands were visualized using Coomassie Brilliant Blue staining.

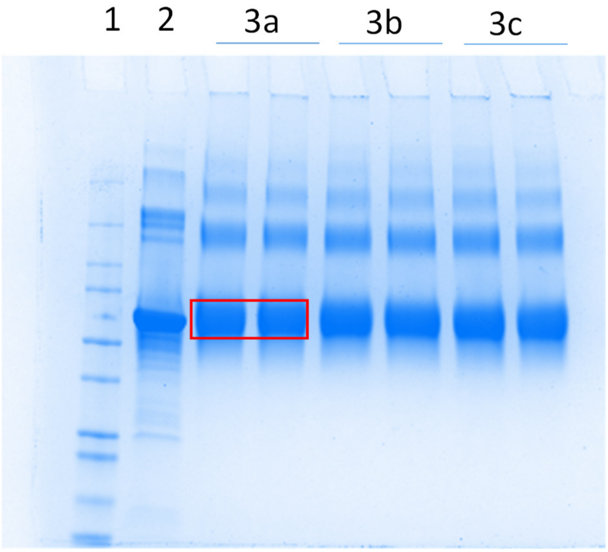

Figure S13. SDS-PAGE analysis of DSBU cross-linking reactions with BSA. Lane 1: molecular weight marker; Lane 2: BSA only; Lanes 3a–3c: replicate cross-linking reactions at a 1:100 molar ratio of BSA to DSBU. Monomeric protein bands (highlighted by red boxes) were excised for in-gel digestion and subsequent mass spectrometry analysis.

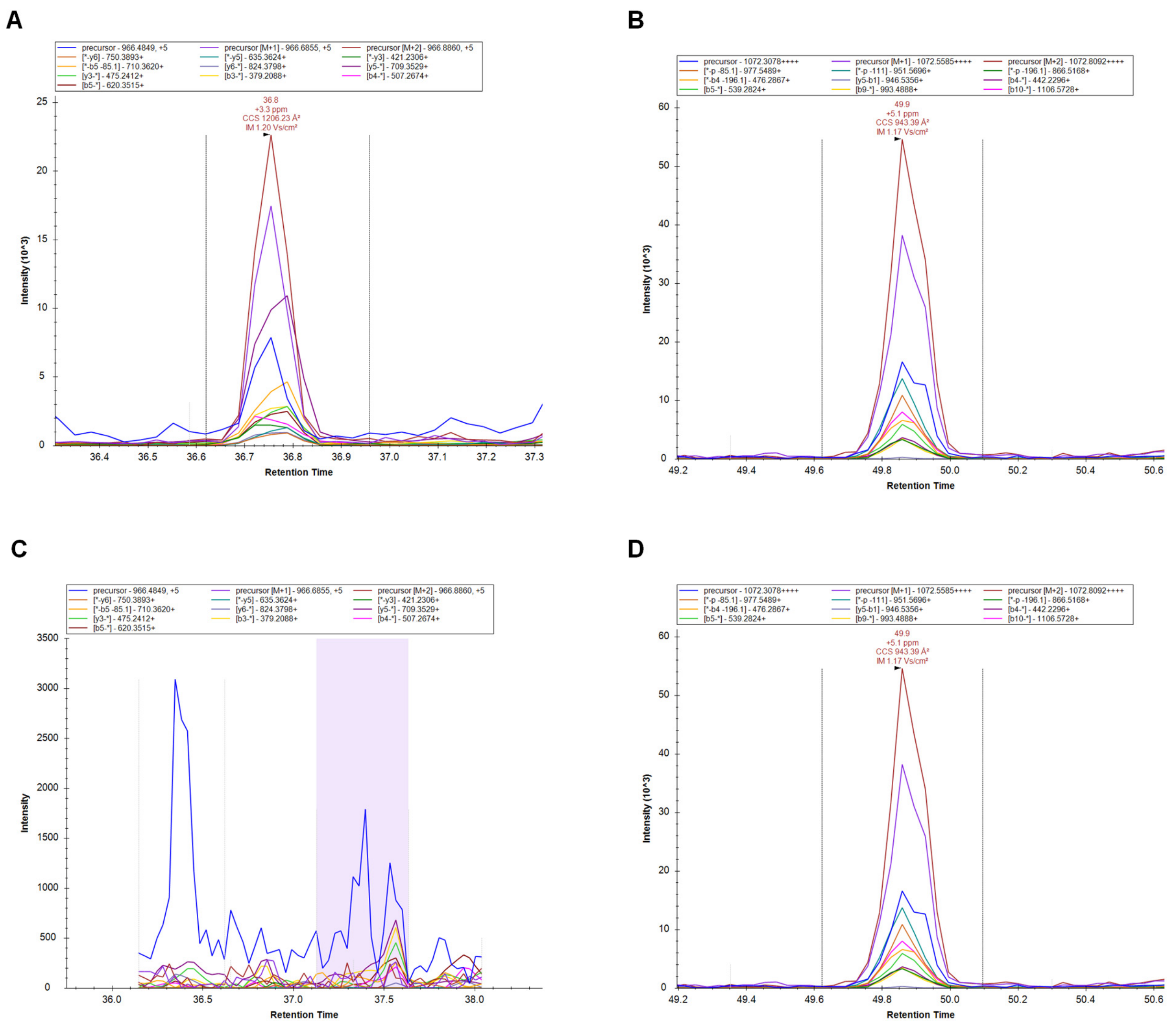

Figure S14. Representative PRM chromatograms of intersubunit NOS–NOS cross-links in wt and E546N iNOS oxyFMN. PRM chromatograms of intersubunit NOS–NOS cross-links 88–696 and 155–607 are shown for dimeric bands of wt (panels A and B) and E546N mutant (panels C and D) iNOS oxyFMN proteins. These cross-links, which occur between the FMN and heme domains, demonstrate both the high quality of the PRM signals and differences in relative cross-link abundance between the two protein variants. The shaded region in panel C indicates a poor match.

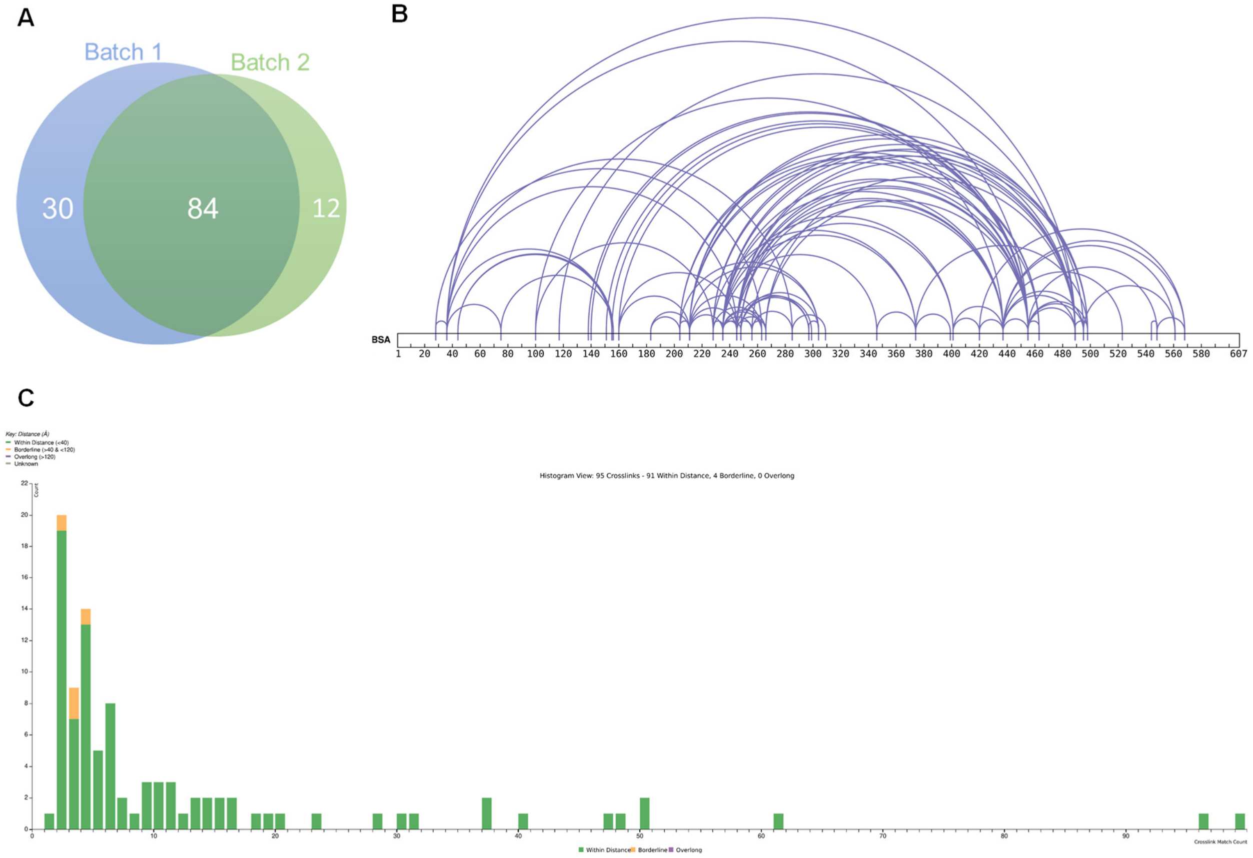

Figure S15. Reproducibility and structural analysis of DSBU cross-links in BSA by timsTOF mass spectrometry. (A) Venn diagram illustrates the overlap of DSBU cross-links identified across replicates of the BSA control protein on different days. (B) Total of 95 intra-BSA cross-links identified within a single batch. (C) Histogram showing the residues' distance distribution of cross-links mapped onto the crystal structure of BSA (PDB ID: 4F5S).

Table S1. Confidence scores for top-ranked AlphaLink2 and AlphaFold 2 structural models of iNOS oxyFMN proteins [a]

Proteins	Modeling type	AlphaLink2 parameters [b]	pLDDT	pTM	iPTM [c]
wt	AlphaLink2	20 25 -1 -1	0.868	0.869	0.868
E546N	AlphaLink2	20 25 -1 -1	0.868	0.757	0.726
		3 25 42 1	0.845	0.877	0.849
wt	AlphaFold2	N/A	0.879	0.864	0.858
E546N	AlphaFold2	N/A	0.882	0.870	0.851

[a] The confidence scores were extracted from the top-ranked (rank001) or best model's PDB and corresponding JSON files using the PAE Viewer (1).

[b] AlphaLink2 default parameters: 20 recycling iterations; 25 structural samples; Neff set to –1; MSAs for crosslinked residues removed by setting values to –1. Additional customizable parameters were evaluated to increase model diversity.

[c] The predicted template modeling (pTM) score (3) and interface predicted template modeling (ipTM) score (4) are based on the template modeling (TM) score, which assesses the accuracy of the global protein structure while being relatively insensitive to localized errors (5). The pTM score reflects the overall accuracy of the predicted protein complex structure, whereas the ipTM score evaluates the accuracy of the relative positioning of the subunits within the complex.

References

1. Elfmann, C., and Stulke, J. (2023) PAE viewer: a webserver for the interactive visualization of the predicted aligned error for multimer structure predictions and crosslinks *Nucleic Acids Res* **51**, W404–W410
2. Bittrich, S., Segura, J., Duarte, J. M., Burley, S. K., and Rose, Y. (2024) RCSB protein Data Bank: exploring protein 3D similarities via comprehensive structural alignments *Bioinformatics* **40**, btae370
3. Jumper, J., Evans, R., Pritzel, A., Green, T., Figurnov, M., Ronneberger, O. *et al.* (2021) Highly accurate protein structure prediction with AlphaFold *Nature* **596**, 583–589
4. Evans, R., O'Neill, M., Pritzel, A., Antropova, N., Senior, A., Green, T. *et al.* (2022) Protein complex prediction with AlphaFold-Multimer *bioRxiv* 10.1101/2021.10.04.463034 %J bioRxiv2021.2010.2004.463034
5. Xu, J., and Zhang, Y. (2010) How significant is a protein structure similarity with TM-score = 0.5? *Bioinformatics* **26**, 889–895
